# Target-dependent biogenesis of cognate microRNAs in human cells

**DOI:** 10.1038/ncomms12200

**Published:** 2016-07-22

**Authors:** Mainak Bose, Suvendra N. Bhattacharyya

**Affiliations:** 1 RNA Biology Research Laboratory, Molecular Genetics Division, CSIR-Indian Institute of Chemical Biology, 4, Raja SC Mullick Road, Kolkata 700032, India

## Abstract

Extensive research has established how miRNAs regulate target mRNAs by translation repression and/or endonucleolytic degradation in metazoans. However, information related to the effect of target mRNA on biogenesis and stability of corresponding miRNAs in animals is limited. Here we report regulated biogenesis of cognate miRNAs by their target mRNAs. Enhanced pre-miRNA processing by AGO-associated DICER1 contributes to this increased miRNP formation. The processed miRNAs are loaded onto AGO2 to form functionally competent miRISCs both *in vivo* and also in a cell-free *in vitro* system. Thus, we identify an additional layer of posttranscriptional regulation that helps the cell to maintain requisite levels of mature forms of respective miRNAs by modulating their processing in a target-dependent manner, a process happening for miR-122 during stress reversal in human hepatic cells.

## Abstract (web summary)

MicroRNAs are a widespread regulatory mechanism and are themselves extensively regulated. Here the authors show regulated miRNA biogenesis by the target mRNA, a layer of regulation that modulates miRNA levels dependent on target availability.

## Graphical abstract

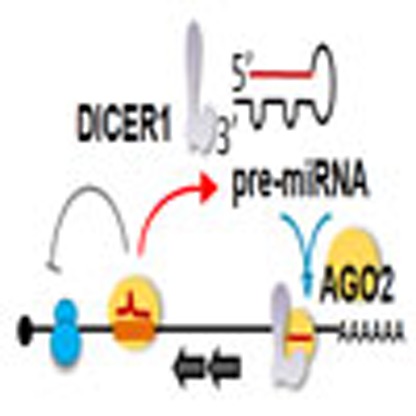

MicroRNAs (miRNAs) are small ∼21-nucleotide-long non-coding RNAs that act as the key posttranscriptional regulators of gene expression in metazoans. In mammals, miRNAs are predicted to control the activity of >60% of all protein-coding genes and participate in the regulation of almost every cellular process investigated to date^1^. Efficient miRNA functioning requires its assembly into miRNPs where the miRNA guide strand serves as the specificity determinant for target RNA recognition and the effector proteins, primarily comprising Argonaute, mediate translation repression and/or target RNA degradation. Animal miRNAs usually hybridize with imperfect complementarities to 3′-untranslated region (UTR) of target messenger RNAs. The 5′-seed region of the small RNA is crucial for target recognition and 3′-half contributes to the stability of the association^2^. The human genome codes for four different Argonaute proteins (hAGO1–4)^3^,^4^. Of these, AGO2 is the most abundantly expressed Argonaute protein^5^. AGO2 is primarily responsible for endonulceolytic cleavage of messages with perfect complementarity to small RNAs^6^.

miRNAs are endogenously transcribed from specific genes by RNA polymerase II as capped and poly-adenylated primary transcripts (pri-miRNAs) that are processed within the nucleus by the Microprocessor complex (Drosha/DGCR8 in humans) to generate 60- to 70-nt-long stem-loop precursor molecules (pre-miRNAs)^7^,^8^,^9^. The pre-miRNAs are exported from the nucleus to the cytoplasm via the Exportin 5 complex^10^,^11^,^12^, where the RNase III endonuclease DICER1 processes these precursors to generate transient double-stranded miRNA/miRNA* duplexes with 2 nt 3′-overhangs^13^,^14^. The exact mechanism of miRNA Induced Silencing Complex (miRISC) assembly has always remained elusive. A human miRNA loading complex (miRLC) has been described, which displays both precursor processing and RISC cleavage activity when exposed sequentially to a miRNA precursor and to a fully complementary target RNA^15^,^16^. Thus, the miRLC couples the process of miRNA biogenesis with target RNA cleavage. The miRLC comprises of DICER1, TRBP2 and miRNA-free AGO protein as its components. Mammalian DICER1 enzymes are large 217 kDa proteins containing ATPase/RNA helicase, DUF283, PAZ domains, two catalytic RNase III domains and a carboxy-terminal dsRBD^17^,^18^. The RNaseIII domain of DICER1 interacts with PIWI domain of AGO proteins, which is crucial for miRNA loading of AGOs^19^. TRBP2, a dsRBD protein partner of DICER1, has been shown to be required for optimal silencing of target gene^20^. Furthermore, it has also been shown that once the AGO2 is loaded with the miRNA, the miRISC dissociates from the miRLC and the loaded miRISC can now catalyse multiple rounds of repression and target RNA cleavage^16^.

More than half of the protein-coding genes in humans contain at least one conserved miRNA binding site apart from several other non-conserved sites^17^. Therefore, it is apparent that biogenesis, function and turnover of these small RNAs need to be effectively regulated. miRNA gene transcription, microprocessor-mediated pri-miRNA processing, exportin-mediated export from the nucleus to cytoplasm and cytoplasmic pre-miRNA processing are all reported to be under stringent regulation^17^. The target mRNA could itself act as a regulator of the miRNA. In *Caenorhabditis elegans*, it has been observed that exoribonuclease XRN-2-mediated degradation of miRNAs is prevented in the presence of target mRNAs^21^. On the contrary, it has been reported in *Drosophila* that extensive complementarities between a target RNA and an Argonaute1-bound miRNA trigger miRNA tailing and 3′–5′ trimming^22^.

We observe target mRNA-dependent biogenesis of mature miR-122 from pre-miR-122 in human hepatoma cells during recovery from amino acids starvation-related stress. This eventually leads to the finding that the presence of abundant amounts of mRNA bearing target sites for a particular miRNA induces increased biogenesis of the mature miRNA from the precursor. These miRNAs are loaded onto AGO2 to form functionally active miRISCs. The increased production of miRNA is proportional to the concentration of target mRNA. Using *in vitro* RISC-loading assay systems, we identify that increased processivity of AGO2-associated DICER1 in the presence of target mRNA contributes to higher biogenesis of mature miRNA from the pre-miRNA.

## Results

### Amino acid stress reversal induces miR-122 biogenesis

In the human hepatoma cell line Huh7, expression of cationic amino acid transporter-1 (CAT-1) is regulated by miR-122 (refs 23, 24). The 3′-UTR of human CAT-1 mRNA harbours four miR-122-binding sites. In Huh7 cells, low CAT-1 protein level is maintained by high miR-122 activity. However, on amino acid starvation CAT-1 mRNA is upregulated^23^. Besides a transcriptional upsurge, repressed CAT-1 mRNAs are released from RNA processing bodies or P-bodies and recruited to polysomes leading to a translational hike^25^. How the excess CAT-1 mRNAs get back to a repressed state when the starved cells are re-exposed to amino acids has not been studied.

**Figure 1:**
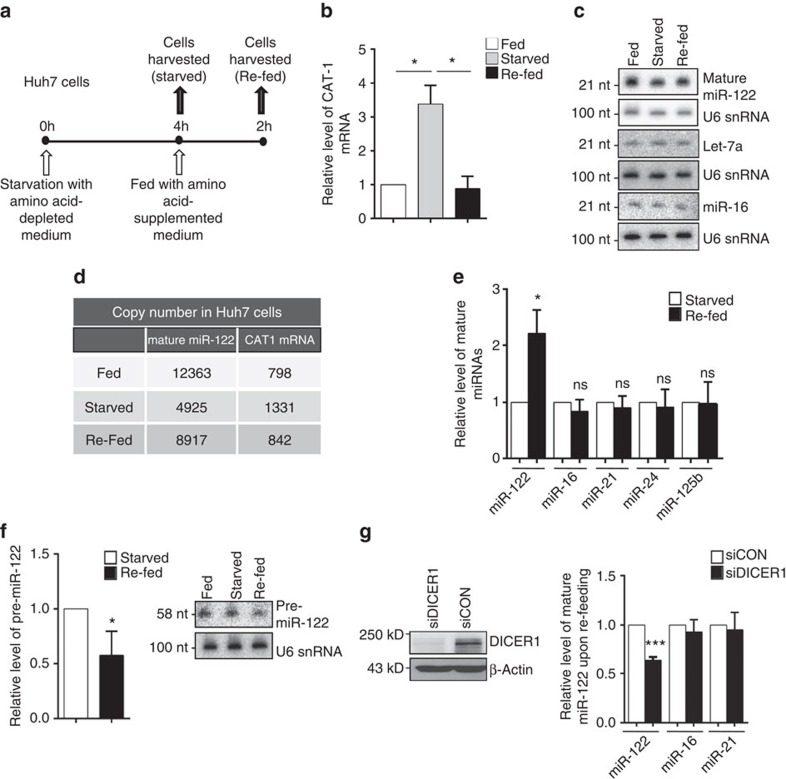
Reversal of amino acid-starvation-induced stress increases miR-122 biogenesis in Huh7 cells. (**a**,**b**) Outline of the experimental set-up used (**a**). Relative level of CAT-1 mRNA in Huh7 cells either fed or starved for 4 h and re-fed with media containing amino acids for another 2 h. CAT-1 mRNA levels were quantified by quantitative reverse transcriptase-PCR with levels in Fed cells taken as 1 (**b**). (**c**) Effect of starvation and re-feeding on mature miR-122 levels in Huh7 cells. Total RNA was extracted and 8 μg RNA was used for northern blotting of mature miR-122, let-7a and miR-16. U6 snRNA was used as loading control. (**d**) Copy number/number of molecules of mature miR-122 and CAT-1 mRNA per Huh7 cell calculated in fed, starved and re-fed Huh7 cells. Estimations were done by real-time-based methods. (**e**) Increase in mature miR-122 but not that of other non-relevant miRNAs, miR-16, miR-21, miR-24 and miR-125b on relief of starvation. Real-time PCR-based quantification of mature miRNA levels in Huh7 cells starved for amino acids (4 h) and subsequently re-fed (2 h). (**f**) Increase in mature miR-122 is accompanied by a concomitant decrease in pre-miR-122 on re-feeding the starved cells for 2 h. Cellular small RNA population was isolated by mirVANA kit to minimize possible contamination of pri-miR-122 and real-time-based assays were carried out to quantify pre-miR-122. Pre-miR-122 detected by northern blotting with 15 μg total RNA. Synthetic pre-miR-122 was used as a size marker to determine the position of the pre-miR-122 in the northern blotting. (**g**) Increase of mature miR-122 on re-feeding of starved cells is reduced in DICER1 knockdown Huh7 cells. miR-16 and miR-21 did not show any significant change. siDICER1-mediated knockdown of DICER1 is confirmed by western blotting. Paired two-tailed Student's *t*-tests were used for all comparisons. *P<0.05, **P<0.01 and ***P<0.001. Error bars represent s.d. (*n*⩾3).

In an attempt to decipher the effect of stress reversal, Huh7 cells were starved for 4 h, then replenished with amino acid-supplemented medium for additional 2 h, and changes in miRNA and CAT-1 mRNA levels were scored (Fig. 1a). Within 4 h of amino acid starvation, there was a threefold surge in CAT-1 mRNA (Fig. 1b). However, on re-feeding the cells with amino acids, within 2 h CAT-1 mRNA was restored to a lower level accompanied by an increase in mature miR-122 level (Fig. 1b,c,e). Quantification of absolute copy numbers of miR-122 and CAT-1 mRNA per cell further confirmed the reciprocal regulation of these two molecules during starvation and re-feeding (Fig. 1d). The rise in miR-122 level is expected, as it would help the cells to restore CAT-1 levels back to normal. We verified that the decrease in CAT-1 mRNA was not due to transcriptional downregulation (Supplementary Fig. 1a). Interestingly, the effect was specific for miR-122 alone, as levels of other miRNAs such as let-7a, miR-16, miR-21, miR-24 and miR-125b were unaffected, arguing against the possibility of a global increase in miRNA levels on stress relief (Fig. 1c,e). The elevated miR-122 was functionally active, as it was AGO2 associated. Consistent with higher miR-122 level, CAT-1 mRNA also showed higher association with AGO2 in re-fed cells (Supplementary Fig. 1b).

Transcriptional upregulation of miR-122 did not contribute to the increased mature miR-122 level, as inhibition of RNA Polymerase II by α-Amanitin could not prevent this increase (Supplementary Fig. 1c). The increase in miR-122 was accompanied by a sharp decrease in pre-miR-122, suggesting increased processing of pre-miR-122 to generate mature miR-122 on stress reversal (Fig. 1f). As DICER1 processes pre-miRNA to the mature form, we suspected a role of DICER1 in the miR-122 surge, but did not detect any increase in DICER1 protein level on stress reversal (Supplementary Fig. 2a). An increase in DICER1 activity could potentially contribute to the observed increase in mature miR-122. However, processing of other miRNAs was unchanged, negating the possibility of global increase in DICER1 activity (Fig. 1e). However, the increase in mature miR-122 on re-feeding the starved cells was reduced on small interfering RNA (siRNA)-mediated knockdown of DICER1, suggesting a requirement of DICER1 in this process (Fig. 1g).

We hypothesized that CAT-1 along with all other stress-induced elevated miR-122 target mRNAs trigger miR-122 biogenesis on re-feeding of starved Huh7 cells. Therefore, if stress-induced upregulation of these mRNAs is prevented, the increase in mature miR-122 levels on stress relief should not occur. To downregulate CAT-1 along with other miR-122 target genes we introduced excess pre-miR-122 before starvation. Under this condition, on relief of starvation, the target-driven miR-122 biogenesis was not observed compared with the control where a nonspecific pre-miRNA was pre-expressed, confirming the role of elevated miR-122 targets in promoting faster miR-122 maturation (Supplementary Fig. 2c).

### mRNAs increase the levels and activity of cognate miRNAs

**Figure 2:**
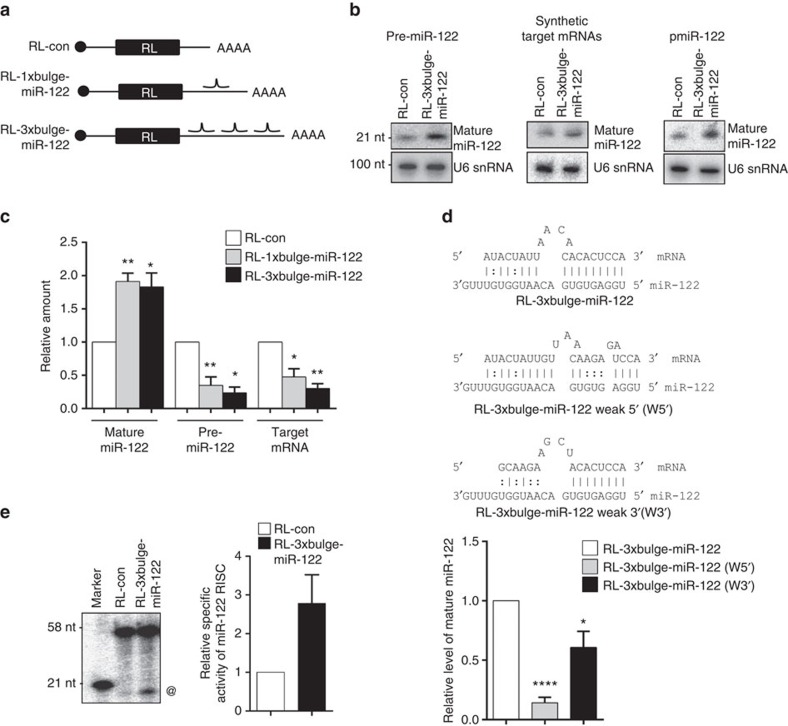
Target mRNA-dependent increase of mature miR-122 in human cells. (**a**) Scheme of the different target mRNAs used. Positions of the miR-122-binding sites are indicated. (**b**) Effect of RL-3 × bulge-miR-122 on mature miR-122 level in cells transfected with pre-miR-122 and reporter plasmids or *in vitro*-transcribed mRNAs. In experiment described in the right panel, HEK293 cells co-transfected with plasmid encoding pre-miR-122 (pmiR-122) and RL reporters were used. Total RNA was extracted and northern blotted for mature miR-122, for all the experiments. U6 snRNA was used as loading control. (**c**) Mature miR-122, pre-miR-122 and target mRNA levels were quantified by quantitative reverse transcriptase–PCR in HEK293 cells expressing target mRNAs and co-transfected with plasmid encoding pre-miR-122. (**d**) Effect of modification of 5′ or 3′ miR-122-binding site on target mRNA-driven miRNA elevation. Relative quantification of mature miR-122 level increase in the presence of target RL-3 × bulge-miR-122 mRNA and in the presence of mRNAs with weak 5′- region (W5′) or weak 3′-region (W3′). Relative levels were normalized against respective target mRNA levels. (**e**) *In vitro* RISC cleavage assay done with protein equivalent amounts of affinity-purified FH-AGO2 isolated from pre-miR-122-transfected FH-AGO2 stable HEK293 cells expressing RL-3 × bulge-miR-122 or RL-con. @, Cleaved product of RISC assay; radio labeled 21-nt band serves as a marker. Paired two-tailed Student's *t*-tests were used for all comparisons. *P<0.05, **P<0.01 and ***P<0.001. Values plotted are means from at least three biological replicates for **c** and **d**, and two biological replicates for **e**. Error bars represent s.d.

To validate whether target mRNA can trigger biogenesis of cognate mature miRNA from their precursors, pre-miR-122 and reporter target mRNAs were co-expressed in HEK293 cells that do not express the liver-specific miR-122. This minimized any ambiguity arising from transcriptional regulation of miR-122 expression in the cell and provided a relatively clean system to study the effect of target mRNA on miRNA levels. An increase in mature miR-122 content was observed in the presence of target mRNAs along with a decrease in the pre-miR-122 level in cells transiently expressing pre-miR-122 along with target mRNA bearing a single-bulged (RL-1 × bulge-miR-122) or three-bulged (RL-3 × bulge-miR-122) miR-122-binding sites in their 3′-UTRs (Fig. 2a–c). Apart from miR-122, levels of other endogenous miRNAs such as let-7a, miR-16 and miR-21 did not change significantly in the presence of the miR-122 targets (Supplementary Fig. 3a). The level of another miRNA, let-7a, also increased specifically on expression of its target mRNA bearing three-bulged let-7a sites (RL-3 × bulge-let-7a) (Supplementary Fig. 3b). It has been shown that the 5′-half of siRNA is essential for target RNA recognition, whereas the 3′-half contributes to the stability of the association^2^. It was observed that the 5′-seed sequence is crucial for target recognition as a seed sequence mutant RL-3 × bulge-miR-122 (W5′) is not repressible by miR-122 (Supplementary Fig. 3d). Interestingly, the 3′-half also binds target mRNA (Supplementary Fig. 3c) and the 3′-sequence mutant RL-3 × bulge-miR-122 (W3′) was less efficiently repressed than the wild-type mRNA (Supplementary Fig. 3d). Altering the 5′-seed-binding sequences on the target mRNA significantly down regulate the target-driven miRNA increase. Tampering with the 3′-half sequence also affected target-driven biogenesis, indicating that both halves contribute to the process (Fig. 2d).

Are these enhanced miRNAs functionally active? *In vitro* RISC cleavage assay with equivalent amounts of affinity-purified AGO2 demonstrated increased miRISC activity in the presence of RL-3 × bulge-miR-122 (Fig. 2e). Further quantification of miR-122 association with affinity-purified AGOs revealed an elevated association of mature miR-122 with all the three Argonautes tested, in a target-dependent manner (Supplementary Fig. 3e). Furthermore, the *in vivo* repressive activity of miR-122 was enhanced in the presence of its target GFP-3 × bulge-miR-122 compared with green fluorescent protein (GFP) control. The effect of target mRNA was specific, as let-7a target mRNA increased let-7a repressive activity without affecting miR-122 activity in co-expressing cells (Supplementary Fig. 3f).

### Target RNA concentration-dependent increase in miRNA level

**Figure 3:**
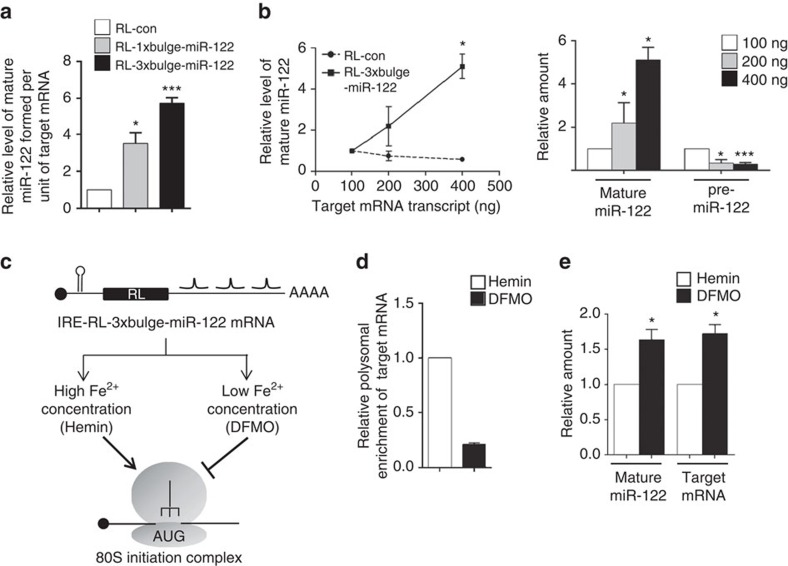
Effect of target mRNA concentration on substrate-dependent miRNA increase in human cells. (**a**) Amount of mature miR-122 formed per unit of target mRNA in HEK293 cells transfected with pmiR-122 and respective reporter plasmids. Values were calculated by normalizing the amount of mature miR-122 against the amount of respective target mRNA level and plotted. (**b**) Effect of increasing concentration of target mRNA on mature miRNA levels. HEK293 cells expressing pre-miR-122 were transfected with increasing amounts of *in vitro*-transcribed mRNA (RL-con or RL-3 × bulge-miR-122) and mature miR-122 and pre-miR-122 levels were quantified 6 h post transfection. In the left panel, changes in relative level of mature miR-122 has been plotted for experiments done with RL-con or RL-3 × bulge-miR-122. Relative change of mature and pre-miR-122 in the presence of different amounts of RL-3 × bulge-miR-122 was plotted (right panel). Values obtained with 100 ng of transcript to transfect 2 × 10^5^ cells were considered as 1. (**c**) IRE-RL-3 × bulge-miR-122 mRNA with Ferritin IRE element in 5′-UTR is schematically depicted. (**d**) Cells transfected with pre-miR-122 and IRE-RL-3 × bulge-miR-122 were split 24 h post transfection and iron chelator DFMO (100 μM) or Fe^2+^ source Hemin (50 μM) was added after an additional 6 h. Cells were harvested after 16 h post Hemin or DFMO addition for analysis. Polysomal enrichment of IRE-RL-3 × bulge-miR-122 was estimated by normalizing polysomal mRNA content by total mRNA level. (**e**) Target mRNA and mature miR-122 level were measured in cells treated with either Hemin or DFMO. Paired two-tailed Student's *t*-tests were used for all comparisons. *P<0.05, **P<0.01 and ***P<0.001. In **a**–**e**, error bars represent s.d. (*n*⩾3).

Expression of RL-1 × bulge-miR-122 induced similar or slightly higher mature miR-122 levels in comparison with RL-3 × bulge-miR-122, despite having a single miR-122-binding site. This was due to higher abundance of RL-1 × bulge-miR-122 mRNA compared with RL-3 × bulge-miR-122 (Fig. 2c). However, the amount of mature miR-122 formed per unit of mRNA was higher for the mRNA with three miRNA-binding sites than with only a single binding site (Fig. 3a). Therefore, the concentration of free miRNA binding sites either placed in *cis* (RL-3 × bulge-miR-122) or in *trans* (RL-1 × bulge-miR-122) regulate target-dependent miRNA biogenesis. To test the effect of target mRNA concentration on the observed miRNA increase, we introduced increasing amounts of synthetic target mRNAs and observed a correlative increase in mature miR-122 levels coupled with a simultaneous reduction in pre-miR122 levels (Fig. 3b). These show that the target-dependent miRNA increase is a function of the concentration of target mRNA. This implies that agents that increase target mRNA levels can promote higher miRNA biogenesis. Cycloheximide, a translation elongation blocker, when applied at a low concentration of 100 ng ml^−1^ diminished translation efficiency^26^ (Supplementary Fig. 4a,b). Cycloheximide-mediated reduced translation led to higher reporter mRNA levels and a corresponding increase in mature miR-122 (Supplementary Fig. 4c).

To investigate how the translatability of the target mRNA may affect substrate-dependent miRNA increase we manipulated the mRNA in various ways so as to alter its translation efficiency. Introduction of a *cis*-regulatory *p27* structural element, in the 5′-UTR of RL-3 × bulge-let-7a reduced mRNA translation efficiency by impaired translation initiation^27^,^28^ (Supplementary Fig. 4d,e). This was accompanied by an increase in the p27-containing target mRNA and increased let-7a levels in HEK293 cells (Supplementary Fig. 4f). The presence of an iron response element (IRE) in 5′-UTR of mRNA regulates initiation as a function of intracellular Fe^2+^ concentration. The binding of iron regulatory protein (IRP) to the IRE sequence inhibits its translation by preventing 40S binding to mRNA^29^,^30^. Under low Fe^2+^ conditions, IRP binds IRE and prevents translation initiation, whereas high Fe^2+^ levels induce higher translation initiation, owing to dissociation of IRP. Introduction of the ferritin IRE in the 5′-UTR of RL-3 × bulge-miR-122 is therefore expected to regulate mRNA translation depending on cellular iron availability (Fig. 3c). Impaired translation in the presence of deferoxamine mesylate salt (DFMO; an iron chelator) was confirmed by the relatively lower retention of the IRE-containing mRNA in the polysomal fractions (Fig. 3d). Reduced translation using DFMO as a Fe^2+^ chelator resulted in higher mRNA levels and higher miR-122 formation (Fig. 3e). Thus, impaired translatability of the mRNA increased the target-driven miRNA production, owing to their higher abundance.

### mRNA increases biogenesis of mature miRNA from pre-miRNA

**Figure 4:**
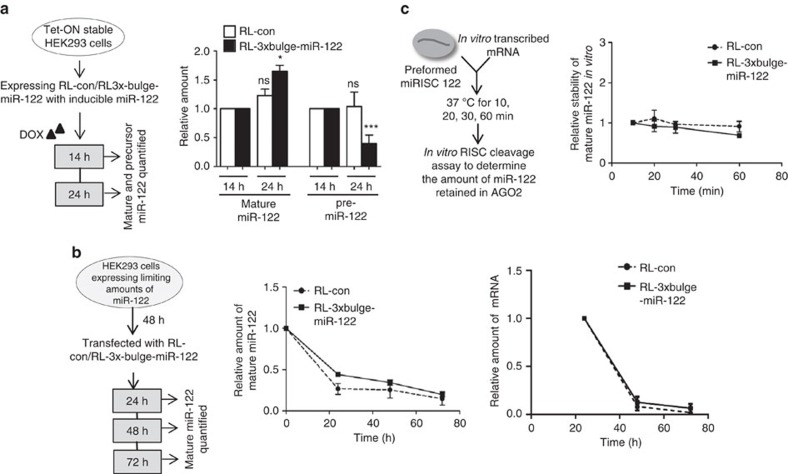
Target mRNA drives increased biogenesis of mature miRNA from pre-miRNA. (**a**) *De novo* synthesis of mature miR-122 in the presence of target mRNA is accompanied by a simultaneous drop in pre-miR-122 level. Experimental format is illustrated in the left panel. Tet-ON HEK293 cells were induced for specific time points with doxycycline to synthesize pre-miR-122 from a plasmid with Tet-response element. Cells were harvested after 14 and 24 h, and mature and pre-miR-122 levels quantified. To measure the relative changes at 24 h, values at 14 h are taken as the unit. (**b**) Target mRNA-induced increase of miRNA levels does not occur due to enhanced stability of a preformed miRNP in the presence of target mRNA. Cells were transfected with 1 μM synthetic pre-miR-122. After 48 h, cells were again transfected with RL-con or RL-3 × bulge-miR-122 plasmids. This was followed by RNA isolation after 24, 48 and 72 h, and mature miR-122 levels quantified to plot the decay rate of mature miR-122. Relative changes in levels of target RNAs over time have been plotted. (**c**) FH-AGO2 was immunoprecipitated from FH-AGO2-stable HEK293 cells transfected with pre-miR-122 plasmid and FH-AGO2 beads corresponding to ∼2 × 10^6^ cells were incubated with 500 ng *in vitro*-transcribed RL-con or RL-3 × bulge-miR-122 mRNA in a 20 μl reaction for increasing time. The supernatant was removed and on-bead RISC cleavage assay was subsequently performed to quantify the amount of miR-122 retained with AGO2 post interaction with target mRNA. Paired two-tailed Student's *t*-tests were used for all comparisons. *P<0.05, **P<0.01 and ***P<0.001. Values plotted are means from at least three biological replicates for **a** and two for **b** and **c**. Error bars represent s.d.

Mechanistically, the target-driven increase in mature miRNA level can stem from either enhanced stability of the existing miRNA or increased biogenesis of the mature form from the precursor. To test this, a doxycycline-inducible system was developed to express pre-miR-122 in HEK293 cells already expressing target mRNA. This would minimize the existence of any preformed miRNP-122 and should serve as a useful tool to monitor and measure *de novo* biogenesis of mature miRNA in the presence of its target. There was a significant increase in the *de novo*-synthesized mature miR-122 with time when RL-3 × bulge-miR-122 was present. This was accompanied by a concomitant decrease in pre-miR-122 levels, indicating a higher rate of mature miR-122 biogenesis from pre-miR-122 in presence of RL-3 × bulge-miR-122 (Fig. 4a). The decay kinetics of mature miR-122 was similar in cells expressing RL-con and RL-3 × bulge-miR-122, suggesting that the presence of target mRNA does not have a stabilizing effect on miR-122 (Fig. 4b). To further check the possible stabilizing effect of target mRNA, we incubated preformed miRNP-122 with target mRNAs for up to 1 h *in vitro.* The mature miR-122 content of miRISC was independent of the presence of target mRNA, arguing against the stabilizing action of target mRNA (Fig. 4c).

### Target mRNA enhances the activity of AGO2-associated DICER1

**Figure 5:**
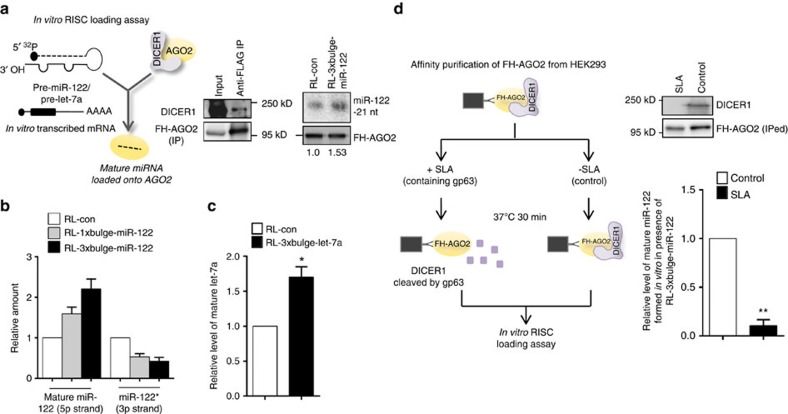
Increased activity of AGO2-associated DICER1 contributes to the target mRNA-driven miRNA production. (**a**–**c**) Increased DICER1 activity in the presence of target mRNA contributes to enhanced miRNA production from pre-miRNA *in vitro.* Scheme of the *in vitro* RISC loading assay has been depicted in the upper panel. Immunoprecipitated FH-AGO2 isolated from HEK293 cells stably expressing the protein was subjected to loading assay with 10 nM pre-miR-122 and 25 ng μl^−1^ of respective target mRNAs. Quantification was done either by densitometry (**a**) or quantitative reverse transcriptase PCR (qRT-PCR) (**b**). The amount of mature miR-122 formed was normalized to the amount of AGO2 immunoprecipitated for quantification. Immunoprecipitation of FH-AGO2 and associated endogenous DICER1 was confirmed by western blotting. Increased DICER1 activity in the presence of target mRNA RL-3 × bulge-let-7a contributes to enhanced miRNA production from pre-let-7a *in vitro* (**c**). (**d**) Removal of AGO2-associated DICER1 impairs target-driven miRNA biogenesis. Scheme of experiment has been shown. Lysate of FH-AGO2-stable HEK293 cells were treated with SLA and FH-AGO2 were immunoprecipitated for *in vitro* loading assay. Quantification was done by qRT-PCR. Paired two-tailed Student's *t*-tests were used for all comparisons. *P<0.05,**P<0.01 and ***P<0.001. For **b**–**d**, values plotted are means from at least three biological replicates. Error bars represent s.d.

To verify whether target mRNA induces higher miRNA biogenesis by faster pre-miRNA processing, we performed an *in vitro* RISC-loading assay with immunoisolated AGO2, synthetic pre-miR-122 and *in vitro* transcribed m^7^G-capped and poly(A)-tailed target mRNAs. The presence of target mRNA enhanced the pre-miRNA processing activity, leading to higher accumulation of mature miR-122 with AGO2 (Fig. 5a). The increased accumulation of mature/5p strand within AGO2 in the presence of miR-122 targets was accompanied by a corresponding decrease in AGO2-bound passenger/3p strand, indicative of successful RISC activation (Fig. 5b)^31^. Higher biogenesis of let-7a in the presence of its target RL-3 × bulge-let-7a was also observed, confirming the generality of target-driven miRNA biogenesis (Fig. 5c). In the *in vitro* pre-miRNA-processing assays with target mRNAs W5′ and W3′, we observed lower target-driven miR-122 biogenesis for W3′ and W5′ target RNA (Supplementary Fig. 5a). During mammalian miRNP assembly by the miRLC, DICER1 processes a pre-miRNA and loads the mature form into a miRNA-free Argonaute^16^. Therefore, it is reasonable that increased activity of AGO-associated DICER1 within the miRLC contributes to the observed increase in miRNA maturation. The role of AGO2-associated DICER1 was confirmed by the observations that knockdown of DICER1 by siRNA (Supplementary Fig. 6) or DICER1 cleavage by soluble Leishmanial antigen (SLA)^32^, containing the metalopretease gp63 that specifically cleave DICER1, resulted in significant reduction of target-driven miRNA biogenesis (Fig. 5d).

**Figure 6:**
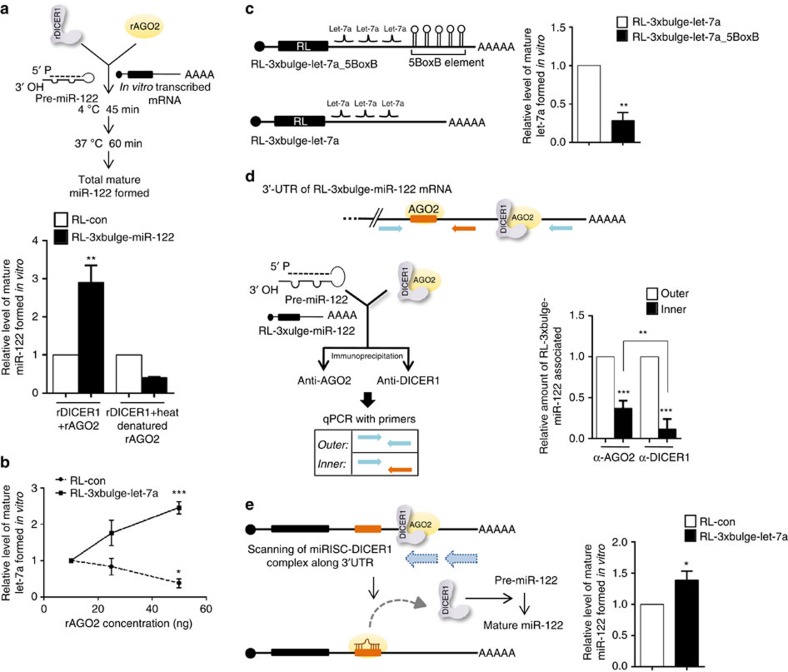
Increased processivity of AGO2-associated DICER1 in the presence of target mRNA. (**a**) *In vitro* pre-miRNA processing assay with rAGO2 and rDICER1 reconfirmed target mRNA-driven increase in AGO2-associated DICER1 activity. *In vitro* pre-miRNA processing assay with rDICER1 and rAGO2 (native or heat-denatured) to quantify miR-122 biogenesis in the presence of target mRNA. Heat denaturation of rAGO2 was carried out at 95 °C for 5 min followed by rapid chilling. (**b**) *In vitro* pre-miRNA processing assay of pre-let-7a with rDICER1 and increasing concentrations of rAGO2 (10, 25 and 50 ng) in the presence of RL-con or RL-3 × bulge-let-7a (25 ng ml^−1^). Mature let-7a levels were measured and plotted. (**c**) Schematic representation of RL-3 × bulge-let-7a_5BoxB mRNA used in the *in vitro* assays. *In vitro* pre-miRNA processing assay of pre-let-7a with rDICER1 and 50 ng rAGO2 in the presence of RL-3 × bulge-let-7a or RL-3 × bulge-let-7a_5BoxB mRNA (both at 25 ng μl^−1^). Mature let-7a levels after the reaction were measured and plotted. (**d**) *In vitro* assay to measure the association of AGO2 and DICER1 along the 3′-UTR of target mRNAs. FH-AGO2 immunoprecipitated from HEK293 cells transiently expressing NHA-DICER1 was subjected to *in vitro* pre-miRNA processing assay with pre-miR-122 and RL-3 × bulge-miR-122 as described earlier, followed by immunoprecipitation of AGO2 and DICER1 with antibodies specific to endogenous proteins. Quantitative reverse transcriptase PCR (qRT-PCR) was done with indicated primers. (**e**) *In vitro* assay to measure processivity of DICER1. Immunopurified AGO2 (let-7a miRISC) incubated with 25 ng ml^−1^ RL-con or RL-3 × bulge-let-7a in the presence of pre-miR-122 (10 nM) at 37 °C for 30 min followed by RNA isolation and quantification of mature miR-122 formed by qRT-PCR. Paired two-tailed Student's *t*-tests were used for all comparisons. *P<0.05, **P<0.01 and ***P<0.001. In **a**–**e**, values are means from at least three biological replicates. Error bars represent s.d.

To validate the contribution of AGO2-associated DICER1 in target-dependent processing of pre-miRNAs we reconstituted the human RISC loading machinery *in vitro* using recombinant AGO2 (rAGO2) with catalytic amounts of recombinant DICER1 (rDICER1). rDICER1 could process the pre-miR-122 *in vitro* but rAGO2 alone failed to process pre-miR-122, eliminating the possibility of enhanced miRNA maturation by increased AGO2 slicer activity^33^ (Supplementary Fig. 5b,c). In assays using rDICER1 and rAGO2, target-dependent biogenesis of miR-122 was observed. However, heat denaturation of rAGO2 impaired the target-driven increase, confirming the importance of AGO2 association of DICER1 in the observed increase in miR-122 biogenesis (Fig. 6a).

It is difficult to envisage how a miRNA-binding site on the mRNA triggers enhanced activity of DICER1 associated with AGO2. DICER1 acts as a multiple-turnover enzyme in the miRLC, wherein it catalyses one cycle of miRNA loading on to associated AGO2, then switches to another free AGO2 molecule to catalyse another cycle of loading^16^. As catalytic activity of DICER1 is directly coupled to AGO2 loading of miRNA, increased DICER1 activity in the presence of target mRNA implies increased AGO2 loading. We hypothesize that immediately after a successful miRNA loading, DICER1 remains associated with AGO2. The complex associates with a mRNA and scans for a target site^2^. Once the target site is found and a stable hybrid is formed, DICER1 dissociates from the miRLC and jumps to a fresh Argonaute for another round of miRNA loading. This step is accelerated when a target mRNA is abundant, thereby contributing to increased processivity of DICER1, which leads to loading of a greater number of Argonaute molecules per unit of time. In support of this model, it was observed that with increasing amounts of rAGO2 and a constant amount of rDICER1, there was a concentration-dependent increase in mature let-7a formation in the presence of RL-3 × bulge-let-7a but not RL-con in an *in vitro* assay (Fig. 6b). Furthermore, it was observed that blocking the mRNA scanning process of loaded AGO2 by introducing a five BoxB hairpin RNA secondary structural element in the 3′-UTR reduces the rate for target site finding and affected target-driven miRNA biogenesis (Fig. 6c and Supplementary Fig. 5d).

We further attempted to verify the importance of increased DICER1 processivity on miRNA processing and loading in an *in vitro* system. According to the proposed model, after scanning along the 3′-UTR, binding of miRISC with the target site should trigger dissociation of AGO2-associated DICER1. Hence, the amount of DICER1 associated with target site (via-AGO2) should be lower compared with its association with the other half of the 3′-UTR. To test this we immunoprecipitated DICER1 and AGO2 after completion of the *in vitro* processing reaction and analysed the 3′-UTR regions that they were directly or indirectly associated with. DICER1 showed relatively less association with the region proximal to target site compared with AGO2, which showed association both with regions distal as well as proximal to miRNA target site regions (Fig. 6d). The processivity of AGO2-dissociated DICER1 during the target-driven miRNA biogenesis was measured by adding a foreign pre-miRNA molecule in the system and scoring its biogenesis. We introduced pre-miR-122 as the precursor of non-relevant miRNA in the *in vitro* assay where RL-con or RL-3 × bulge-let-7a was incubated with let-7a miRISC. It was observed that the amount of mature miR-122 formed was higher in the presence of the let-7a target mRNA compared with that of RL-con (Fig. 6e).

## Discussion

We have documented that target mRNA triggers increased miRNA production and activity in HEK293 cells. As it is difficult to identify the exact reason for the observed increase in miRNA activity in cellular context, we wanted to recapitulate the observation *in vitro*. Evidences from *in vitro* studies suggested that increase in DICER1-mediated pre-miRNA processing is responsible for the target-driven increase in miRNA biogenesis. Notably, the target mRNA-induced miRNAs are loaded into AGO proteins to form active miRNPs.

**Figure 7:**
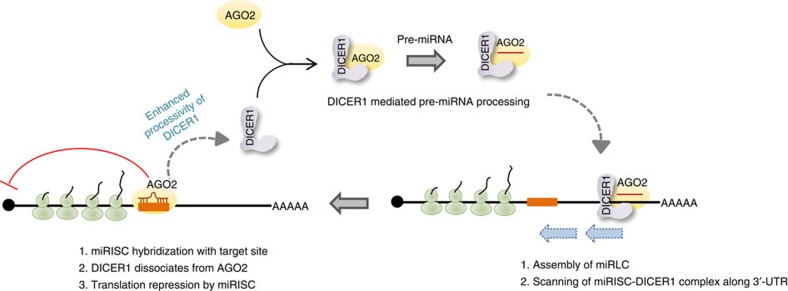
A model of target-driven miRNA biogenesis. Schematic model of target mRNA-driven miRNA biogenesis. Immediately after pre-miRNA processing and AGO2 loading, DICER1 remains associated with AGO2. Newly formed miRISC/DICER1 complex scans the 3′-UTR of a mRNA in search of cognate miRNA-binding site. On target site finding and miRNA–mRNA hybrid formation, DICER1 dissociates from AGO2 and binds free AGO2 to catalyse another round of pre-miRNA processing and miRNP formation. The presence of the target sites increases the ‘processivity' of DICER1, leading to enhanced miRNA biogenesis from precursor per unit time.

Gregory *et al*.^15^ have shown a ‘physical and functional coupling' between pre-miRNA processing by DICER1 and loading of AGO2 to assemble a functional RISC. They observed that target RNA cleavage is ten times more efficient when pre-miRNA, a DICER1 substrate, is used compared with a miRNA duplex. As DICER1 catalytic activity is directly coupled to RISC loading, the target mRNA-induced increase in DICER1 activity that we observe here leads to increased AGO loading. The role of DICER1 as a multiple turnover enzyme during RISC loading is established. Our work proposes that the miRLC does not dissociate immediately after AGO2 has been loaded. Instead, it scans the mRNA and once a target site is found and stable miRNP binding occurs, DICER1 dissociates and catalyses another cycle of miRNA loading. As DICER1-mediated AGO loading is processive, the processivity of the enzyme is enhanced when target mRNA is abundant, contributing to higher number of successful loading cycles per unit time (Fig. 7).

The exact mechanism of miRNA passenger strand removal during AGO2 loading has not been resolved. Apart from the slicer-dependent mechanism of miRNA* strand cleavage^31^,^34^, a slicer-independent mechanism has been recognized that plays a more prominent role in loading of miRNAs in mammalian AGOs^35^. Our model supports the second mechanism whereby both the sense and anti-sense strand may remain associated with AGO2 post loading; in case of increased local abundance of target sites, the sense strand hybridizes to target sites and therefore the removal of passenger strand is more efficient (Fig. 5b). In fact, it has been proposed in a recent report that in siRNA-mediated RNA interference, product release from AGO2 is accelerated in the presence of excess target RNA^36^; this can be accounted for by target RNA-dependent enhancement of RISC activity.

Turnover of miRNAs helps the cell to maintain a steady-state level of miRNPs. In certain cell types such as neurons, where dynamic changes in gene expression occur, 3′-tailing of miRNAs with non-templated nucleotides, 3′–5′ trimming and rapid miRNA degradation have been reported^37^. Trimming and tailing have also been reported in *Drosophila*^22^. Interestingly, this degradation was shown to be induced by highly complementary target mRNAs. Several other articles have also reported miRNA degradation induced by highly complementary targets, a phenomenon referred to as target RNA-directed miRNA degradation (TDMD)^37^,^38^,^39^. Apparently, our observations seem to contradict these findings. However, it should be noted that where TDMD refers to turnover of preformed mature miRNAs by complementary target mRNAs, we report increased *de novo* biogenesis of miRNPs by target mRNAs. In the cellular context, one phenomenon can be masked by the other and due to unavailability of any inhibitor of TDMD it is difficult to factually distinguish between the two processes. Therefore, we resorted to an *in vitro* set-up with recombinant proteins and synthetic RNAs, to nullify any cellular event that might complicate our observation. Contrary to TDMD, Chatterjee *et al*., reported that complementary target mRNAs prevent degradation of mature miRNAs in *C. elegans*. Again, this process also depicts the fate of mature preformed miRNAs being regulated by target mRNAs^21^.

We have provided evidence of target-driven miRNA biogenesis using Huh7 hepatoma cells in which, on stress reversal, increased levels of CAT-1 mRNA induced miR-122 production (Fig. 1). Starved Huh7 cells harbour a huge repertoire of CAT-1 mRNA. Hence, it is probable that CAT-1 can trigger increased miR-122 biogenesis in an attempt to restore cellular homeostasis on reversal of stress induced by amino-acid starvation. However, as CAT-1 is one of the numerous miR-122 target mRNAs that surge in starved cells and is responsible for miR-122 increase in re-fed cells, siRNA-mediated knockdown of CAT-1 could only partially reduce the miR-122 level in re-fed cells (Supplementary Fig. 2b). This acts as a feedback mechanism whereby a target mRNA can autoregulate its own levels by driving synthesis of its cognate miRNA. In fact, a miRNA autoregulatory circuit has been reported to operate in *C. elegans*, whereby mature let-7 miRNP binds and induces increased processing of let-7 primary transcripts^40^. There can be a host of other such cellular situations where such a mechanism can regulate miRNA loading and consequently fine tune gene expression. During miRNA biogenesis one strand of the precursor hairpin is selected as the guide strand, whereas the other strand (miRNA*) is degraded. This ‘strand bias' theory has been formulated based on the relative thermodynamic stability of the two sister strands (5p and 3p) in the pre-miRNA; the strand with lesser stability is preferentially selected as guide, whereas the other strand is degraded^41^. Examination of the thermodynamic stability profiles of the pre-miRNA and mature miRNA revealed most of the miRNAs are highly asymmetric with 5p as the guide strand, with only a handful of miRNAs showing comparable stability of 5p and 3p strands^41^,^42^. However, Ro *et al*.^43^ observed that selective or simultaneous accumulation of both strands occur in a tissue-specific manner. This implies that the two opposite strands can target completely different sets of target mRNAs^43^. High-throughput miRNA sequencing analysis has revealed that the arm from which mature miRNA is generated can switch at different developmental stages or in different tissues^44^. A study showed that miR-142-5p was sequenced more frequently in the ovary, testes and brain, and miR-142-3p was sequenced more recurrently in embryonic and newborn samples^17^. Expression of certain mRNAs is tissue specific or developmental stage specific. Therefore, it is probable that the expression of a new mRNA at a particular developmental stage will induce synthesis of cognate miRNA (say 3p of a pre-miRNA); another mRNA in a subsequent developmental stage can induce 5p of the same pre-miRNA. Thus, target mRNA-driven miRNP biogenesis can act as the molecular switch that can trigger selective expression of a particular miRNA strand, thereby acting as a tool to configure gene expression by directing miRNA arm switching.

The 3′-UTR of most of the endogenous target mRNAs bear binding sites for several miRNAs and one or more sites for a single type of miRNA. Therefore, repression of a mRNA by a miRNA can lead to biogenesis of a secondary miRNA that also has a binding site on the same mRNA. This in turn will lead to repression of a different mRNA that has sites for this secondary miRNA. If both genes are members of the same cellular pathway, then repression of one gene can influence expression of another gene. This can act as a plausible mechanism of coordinating gene expression by miRNAs, further supporting the role of these small RNAs as ‘fine-tuners' of gene expression. Importantly, our findings reflect another layer of dynamicity in miRNAs action, whereby cells can maintain only requisite levels of specific miRNPs depending on target availability. Rather than transcriptionally upregulating miRNA synthesis, modulating the final step of biogenesis serves as an immediate means to sense target RNA increase and subsequent enhancement of miRNA production. This in turn helps the cell respond to specific and urgent cellular needs.

## Methods

### Cell culture and transfections

Both Huh7 and HEK293 cells were obtained from the laboratory of Witold Filipowicz. The cells were free of mycoplasma contamination and were cultured in DMEM medium containing 2 mM L-glutamine and 10% heat-inactivated FCS as described earlier^25^,^31^,^33^. All cell culture reagents were from Life Technologies. All plasmid transfections were carried out with Lipofectamine 2000 and siRNAs with RNAiMax (Life Technologies) using the manufacturer's instructions.

In HEK293 cells, 1 μg target mRNA encoding plasmids were transfected in six-well format with 5 pmol pre-miR-122 (Life Technologies) or 500 ng pre-miR-122 encoding plasmid. Four hundred nanograms of synthetic *in vitro*-transcribed target mRNAs were transfected in a 24-well format, unless described otherwise. Transcription blockage was done with 10 μg ml^−1^ α-Amanitin (Calbiochem). For experiments using IRE constructs, 50 μM Hemin (Sigma) or 100 μM DFMO (Calbiochem) was added to cell culture media for desired time. For experiments using Tetracycline-inducible constructs, TET-ON HEK293 cells with the inducble expression constructs were cultured in DMEM supplemented with 10% TET-approved FCS (Clontech) and induction of expression of respective gene product was carried out for indicated time durations using 300 ng ml^−1^ Doxycycline (SIGMA)^47^. 

siDICER1 were transfected at 30 nM concentration in a 24-well format and siCAT-1 at 50 nM in the same format. CAT-1 siRNA (5′- CCAGCCUUAUAGCUGUUCU -3′) was purchased from Eurogentec. Dharmacon SMARTpool ON-TARGETplus siRNAs against DICER1 were purchased from Thermo Scientific. All the plasmids used for the study have been listed in Supplementary Table 1.

### Huh7 starvation and feeding

For starvation experiments, Huh7 cells grown in normal DMEM were incubated in Hanks' balanced salt solution (HBSS) supplemented with 10% dialysed FCS for 4 h followed by ‘re-feeding' cells with HBSS supplemented with 10% normal FCS for an additional 2 h. HBSS supplemented with 10% normal FCS was used for ‘fed' control.

### Luciferase assay

Renilla luciferase (RL) and Firefly luciferase (FF) activities were measured using a Dual-Luciferase Assay Kit (Promega), following the manufacturer's instructions, on a VICTOR X3 Plate Reader with injectors (Perkin Elmer). The ratio of Firefly luciferase normalized Renilla luciferase expression levels for control is to reporter were used to calculate fold repression.

In the experiment using GFP target reporters, HEK293 cells were transfected in a 24-well format with 250 ng pmiR-122, 100 ng firefly luciferase, 500 ng of GFP-con or GFP-3 × bulge-miR-122 along with 10 ng of luciferase reporter RL-con or RL-3 × bulge-miR-122, or RL-3 × bulge-let-7a (additional control). After 24 h cells were split and at 48 h luciferase activity measured. Firefly normalized RL values were plotted. For GFP-3 × bulge-let-7a reporter, exactly the same transfections were performed with an exception of the GFP-3 × bulge-miR-122 reporter.

### RNA isolation and northern blotting

Total RNA was isolated using TRIzol reagent (Life Technologies). Small RNA was isolated using mirVANA miRNA isolation kit (Life Technologies) according to the manufacturer's instructions. Northern blotting of total cellular RNA (5–10 μg) was performed as described by Pillai *et al*.^48^. In short, equal amount of total RNA was electrophoresed in 15% Urea–PAGE followed by elecrtrophoretic transfer to nylon membrane. For detection, γ^32^P-labelled 22-nt miRCURY complementary LNA probes for miR-122 and let-7a (Exiqon) or complementary DNA probe for miR-16, U6 snRNA were used. PhosphorImaging of the blots was performed in Cyclone Plus Storage Phosphor System (Perkin Elmer). Uncropped versions of all blots are given in Supplementary Fig. 7.

### Real-time PCR

Real-time (reverse transcriptase) PCR for mRNA was done with the Mesa Green qPCR Mastermix Plus for SYBR Assay-Low ROX (Eurogentec) by using the 100–200 ng of the total RNA with the primers listed in Supplementary Table 2. The endogenous control used was 18S ribosomal RNA. For the quantification of miRNA, 30 ng of total RNA was taken. Following primers were used for the amplifications of different miRNAs. For example, human let-7a (assay ID 000377), human miR-122 (assay ID 000445), human miR-122* (assay ID 002130), human miR-16 (assay ID 000391), human miR-21 (assay ID 000397), human miR-24 (assay ID 000402), human miR-125b (assay ID 000449), U6 snRNA (assay ID 001973) and Applied Biosystem Taqman chemistry-based miRNA assay system were used for the experiment. All reactions were done in 7500 Applied Biosystem Real Time System or BIO-RAD CFX96 Real-Time system. Cycles were set as per the manufacturer's protocol.

### Copy number determination

To determine copy number of miR-122, we generated a standard curve using PAGE-purified synthetic miR-122. Five nanograms of total RNA from HEK293 cells (does not contain miR-122) were spiked with 10^6^–10^10^ molecules of synthetic miR-122 and analysed by real-time PCR using Taqman miRNA assays (Life Technologies). No Ct was obtained in a control where synthetic miR-122 was not added. The Ct values obtained were plotted to get the standard curve. Ct-values obtained from fed, starved or re-fed samples using 5 and 25 ng of Huh7 RNA were converted to molecule copy number using the standard curve. To calculate copy number of miR-122 per Huh7 cell, we determined total amount of RNA obtained per Huh7 cell under fed, starved and re-fed conditions by again generating a standard curve of total RNA versus number of Huh7 cells. To do that, we counted cells using haemocytometer and isolated RNA by TRIzol reagent and quantified total RNA using NanoDrop. The values obtained were as follows: 34.69 pg (fed 4 h), 21.55 pg (starved 4 h) and 23.19 pg (re-fed 2 h). This value was fairly consistent with Chang *et al*.^24^ who obtained 25 pg of RNA per cell. The copy number obtained by our real-time-based method for non-starved Huh7 (fed) was in that order of 10^4^ (∼1.2 × 10^4^). This was lower than that obtained by Siegrist *et al*.^49^, (3 × 10^4^) using bead array technology.

Similarly, for CAT-1 mRNA standard curve was generated using 100 ng RAW264.7 (mouse cell line) RNA spiked with 10^6^–10^10^ molecules of *in vitro*-transcribed RL-CatA mRNA that contains *Renilla*-coding region fused to the 3′-UTR (bases 2,169–4,646) of human CAT-1 mRNA. A minus RL-catA control yielded no amplification 3′-UTR-specific primers were used and the same set of primers were used to quantify CAT-1 from 500 ng RNA of Huh7 cells.

### Immunoprecipitation

Immunoprecipitation of proteins was done essentially as per published protocols^25^,^50^. Cells were lysed in a lysis buffer [20 mM Tris-HCl pH 7.4, 200 mM KCl, 5 mM MgCl_2_, 1 mM dithiothreitol (DTT), 1 × EDTA-free protease inhibitor (Roche), 5 mM Vanadyl ribonucleoside comples (Sigma), 0.5% Triton X-100, 0.5% sodium deoxycholate] at 4 °C for 15 min, followed by clearing the lysate at 3,000 *g* for 10 min. Protein G agarose beads (Invitrogen) were blocked with 5% BSA in lysis buffer for 1 h and then incubated with required primary antibody for another 3–4 h before the lyaste was added. A final dilution of 1:50 (antibody:lysate) was used for immunoprecipitation. Immunoprecipitation was carried out for 16 h at 4 °C. Post washing with IP buffer (20 mM Tris-HCl pH 7.4, 150 mM KCl, 5 mM MgCl_2_, 1 mM DTT, 1 × EDTA-free protease inhibitor (Roche)), the beads were divided in two halves: one subjected to RNA isolation with TRIzol LS and another for western blotting.

### Immunoblotting

Western blotting of proteins was essentially performed as described previously^48^. For immunoblotting, the cell lysates or immunoprecipitated proteins were subjected to SDS–PAGE, transferred to nitrocellulose membrane and probed with specific antibodies. Antibodies used were as follows: anti-AGO2 (Novus Biologicals; Cat# H00027161-M01; 1:1,000), anti-DICER (Bethyl; Cat# A301-936A; 1:5,000), anti-HA (Roche; Cat# 11867431001; 1:1,000), β-Actin (Sigma; Cat# A3854; 1:10,000) and phospho-eIF2α (Cell Signaling Technology; Cat# 9721; 1:500). Imaging of all western blottings was done in UVP BioImager 600 system equipped with VisionWorks Life Science software (UVP) V6.80 and quantification of bands done using the same software. Uncropped versions of all blottings along with molecular-weight marker positions are included in Supplementary Fig. 7.

### Polysome isolation

Total polysome isolation was carried out as described^51^. For total polysome isolation, around 6 × 10^6^ HEK293 cells were lysed in a buffer containing 10 mM HEPES pH 8.0, 25 mM KCl, 5 mM MgCl_2_, 1 mM DTT, 5 mM vanadyl ribonucleoside complex, 1% Triton X-100, 1% sodium deoxycholate and 1 × EDTA-free protease inhibitor cocktail (Roche) supplemented with Cycloheximide (100 μg ml^−1^; Calbiochem). The lysate was cleared at 3,000 *g* for 10 min followed by another round of pre-clearing at 20,000 *g* for 10 min at 4 °C. The clarified lysate was loaded on a 30% sucrose cushion and ultracentrifuged at 100,000 *g* for 1 h at 4 °C. The non-polysomal supernatant was collected from the top. The sucrose cushion was washed with a buffer (10 mM HEPES pH 8.0, 25 mM KCl, 5 mM MgCl_2_, 1 mM DTT), ultracentrifuged for additional 30 min and the polysomal pellet was finally resuspended in polysome buffer (10 mM HEPES pH 8.0, 25 mM KCl, 5 mM MgCl_2_, 1 mM DTT, 5 mM vanadyl ribonucleoside complex, 1 × EDTA-free protease inhibitor cocktail) for RNA isolation.

### RISC cleavage assay

RISC cleavage assay was essentially performed as described elsewhere with minor modifications^50^,^51^. HEK293 cells stably expressing FH-AGO2 were transfected with pmiR-122 and target mRNA expression plasmids. For miRISC isolation, cells were lysed, 48 h after transfection, in lysis buffer [10 mM HEPES pH 7.4, 200 mM KCl, 5 mM MgCl_2_, 1 mM DTT, 1 × EDTA-free protease inhibitor (Roche), 5 mM vanadyl ribonucleoside comples (Sigma), 1% Triton X-100] at 4 °C for 15 min, followed by clearing the lysate at 3,000 *g* for 10 min. The clarified supernatant was subjected to immunoprecipitation with anti-FLAG M2 affinity gel (Sigma) for 16 h at 4 °C. Beads were washed in IP buffer [20 mM Tris-HCl pH 7.4, 150 mM KCl, 5 mM MgCl_2_, 1 mM DTT, 1 × EDTA-free protease inhibitor (Roche)] for three times at 4 °C. The affinity-purified miRISC was eluted from the beads using 3 × FLAG Peptide (Sigma) as per the manufacturer's instructions in a RISC purification buffer (30 mM HEPES pH 7.4, 100 mM KCl, 5 mM MgCl_2_, 0.5 mM DTT, 3% glycerol).

Affinity-purified miRISC-122 were assayed for target RNA cleavage using a 36 nt RNA 5′- AAAUUCAAACACCAUUGUCACACUCCACCAGAUUAA -3′ bearing the sequence complementary to mature miR-122. Target RNA cleavage assays were carried out in a total volume of 30 μl with 10 fmol of 5' ^32^P-labelled RNA in an assay buffer (100 mM KCl, 5.75 mM MgCl_2_, 2.5 mM ATP, 0.5 mM GTP) and protein equivalent amounts of RISC at 30 °C for 30 min. RNA isolation was carried out from the reaction mixture, cleaved products were analysed on a 12% denaturing 8 M Urea–PAGE and visualized by autoradiography.

### *In vitro* RISC loading assay

FH-AGO2 affinity purified on agarose beads from FH-AGO2-stable HEK293 cells was incubated with 10 nM synthetic 5′-phosphorylated pre-miR-122 or pre-let-7a, and 500 ng *in vitro*-transcribed target mRNA in a 20-μl reaction in assay buffer [20 mM Tris-HCl pH 7.5, 200 mM KCl, 2 mM MgCl_2_, 5% glycerol, 1 mM DTT, 40 U RNase inhibitor (Fermentas)] for 1 h at 37 °C followed by washing of the beads three times with IP buffer. Each reaction was then divided into two halves: one for RNA isolation and the other for western blotting of AGO2.

For assays with recombinant proteins, 50 ng of rAGO2 (Sino Biologicals) was used with 0.1 U of rDICER1 (Recombinant Human Dicer Enzyme kit, Genlantis) keeping the rest of the reaction parameters same as above.

To measure the association of AGO2 and DICER1 along the 3′-UTR, FH-AGO2 stably expressing HEK293 cells was transfected with NHA-DICER1 transiently and FH-AGO2 immunoprecipitated with anti-FLAG agarose beads as described before. FH-AGO2 was eluted from anti-FLAG beads with 3 × FLAG Peptide (Sigma) and isolated miRISC was incubated with RL-3 × bulge-miR-122 at 37 °C with 10 nM pre-miR-122 for 60 min. The reaction was then divided in two halves and immunoprecipitated with anti-AGO2 and anti-DICER1 antibodies for 3 h at 4 °C. Beads were washed and RNA isolated using TRIzolLS followed by quantitative reverse transcriptase–PCR using a single forward and two sets of reverse primers.

For scoring DICER1 processivity, HEK293 cells transiently expressing FH-AGO2 and pre-let-7a were lysed and FH-AGO2 immunoprecipitated as described earlier. FH-AGO2 was eluted from anti-FLAG beads with 3 × FLAG Peptide (Sigma) and isolated miRISC was incubated with RL-con or RL-3 × bulge-let-7a at 37 °C with 10 nM pre-miR-122 for 30 min. RNA was isolated from the reaction and mature miR-122 formed quantified by real-time PCR.

### *In vitro* transcription

*In vitro* transcription was carried out using mMESSAGE mMACHINE kit (Life Technologies) and poly A tailing of transcripts done with Poly (A) tailing kit (Life Technologies) as per the manufacturer's protocol. For preparation of plasmid DNA template, RL-con, RL-1 × bulge-miR-122, RL-3 × bulge-miR-122, RL-3 × bulge-miR-122 (W5′), RL-3 × bulge-miR-122 (W3′) and RL-CatA were digested for 4 h at 37 °C with DraI (New England Biolabs) and RL-3 × bulge-let-7a was digested with HpaI (New England Biolabs); the rest of the protocol was as per the manufacturer's instructions. All transcripts obtained were analysed and size verified by 6% Urea–PAGE and ethidium bromide-based detection.

### RNase protection assay

RNase Protection Assay was performed as described earlier^52^. The synthetic miR-122 RNA probe was 5′-end labelled with γ^32^P ATP using T4 Polynucleotide kinase (Ferementas) according to the manufacturer's protocol. Labelled miR-122 (1.5 pmol; 100 fmol radiolabelled and 1.4 pmol 5′-end labelled with cold ATP) was mixed with 500 fmol of target mRNA and precipitated with 0.1 vol sodium acetate (pH 5.2) and 2.5 vol ice-cold ethanol for 1 h at −80 °C. The RNA recovered was dissolved in 30 μl Hybridization Buffer (40 mM PIPES pH 6.8, 1 mM EDTA pH 8.0, 0.4 M NaCl, 80% deionized formamide), denatured at 85 °C for 5 min and then annealed at 35 °C overnight. The mixture was next cooled to room temperature and then RNase digestion was carried out in 300 μl of RNase digestion mixture (300 mM NaCl, 10 mM Tris pH 7.4, 5 mM EDTA pH 7.5, 30 μg RNase A (Fermentas)) at 30 °C 1 h. ProteinaseK treatment was done (10% SDS and 20 mg ml^−1^ Proteinase K (Roche) at 37 °C for 30 min) followed by RNA extraction with acid phenol:chloroform pH 4.5 in the presence of 20 μg carrier transfer RNA (Sigma). The samples were run in an 18% Urea–PAGE followed by drying the gel and image was obtained using phosphorimager. For partial digestion, the reaction was incubated on ice instead of 30 °C.

### Nuclear run-on transcription

Nuclear run-on transcription was performed as described by Roberts *et al*.^53^. For isolation of intact nucleus from Huh7 cells, ∼4 × 10^6^ cells were lysed in NP-40 lysis buffer [10 mM Tris-HCl pH 7.4, 10 mM NaCl, 3 mM MgCl_2_ and 0.5% (vol/vol) NP-40]. Fractionation verified by western blotting for nuclear and cytosolic markers. The nuclei were resuspended in 30 μl Nuclei Storage Buffer [50 mM Tris-HCl pH 8.3, 0.1 mM EDTA, 5 mM MgCl_2_, 40% (vol/vol) glycerol] and subjected to run-on transcription in transcription buffer (10 mM Tris-HCl pH 8.3, 2.5 mM MgCl_2_, 150 mM KCl, 2 mM DTT, 1 mM ATP, 1 mM CTP, 1 mM GTP, 1 mM GTP, 100 U RNase inhibitor) at 30 °C for 30 min. Immediately after the reaction, RNA was isolated using TRIzolLS and subjected to DNaseI (New England Biolabs) treatment at 37 °C for 15 min. DNaseI was inactivated by heating at 75 °C for 5 min. Six hundred nanograms of RNA was used for cDNA preparation in a 10-μl reaction. Minus reverse transcriptase control was done to verify the absence of any genomic DNA contamination.

### SLA preparation

SLA was prepared using published protocol^54^. Briefly, for SLA preparation, around 10^10^ *Leishmania donovani* cells were washed thrice with 1 × PBS followed by five to six cycles of liquid nitrogen freeze thawing. The cells were then sonicated in PBS with 0.04% NP-40 for six times. Lysate was cleared at 12,000 *g* for 10 min at 4 °C and the supernatant collected was SLA. Protein estimation of SLA was done using Bradford method at 595 nm.

The *in vitro* DICER1 cleavage assay was carried out as mentioned elsewhere^32^. Lysate of HEK293 cells stably expressing FH-AGO2 were prepared as for immunoprecipitation. For each cleavage reaction, 30 μg cell lysate was incubated with 20 μg SLA in assay buffer (10 mM Tris-HCl pH 7.5, 1 mM DTT, 100 mM KCl, 1 × EDTA-free protease inhibitor (Roche)) and 1 mg ml^−1^ BSA for 30 min at 37 °C. This was followed by immunoprecipitation of the mixture with anti-FLAG M2 agarose beads. Post immunoprecipitation, the beads were subjected to *in vitro* DICER activity assay.

### Statistical analysis

All graphs and statistical analyses were done in GraphPad Prism 5.00 (GraphPad, San Diego, CA, USA). Nonparametric paired *t*-test were used for analysis and *P*-values were determined. Error bars indicate mean±s.d.

### Data availability

The authors declare that all data supporting the findings of this study are available within the article and its Supplementary Information files or on request.

## Additional information

**How to cite this article:** Bose, M. and Bhattacharyya, S. N. Target-dependent biogenesis of cognate microRNAs in human cells. *Nat. Commun.* 7:12200 doi: 10.1038/ncomms12200 (2016).

## Supplementary Material

Supplementary Information Supplementary Figures 1-7, Supplementary Tables 1-2 and Supplementary References
