# Supplementary material for: Target-dependent biogenesis of cognate microRNAs in human cells
Source: Nat Commun. 2016 Jul 22;7:12200. doi: 10.1038/ncomms12200 (PMC4961841; doi:10.1038/ncomms12200)
Supplement: Supplementary Information — Supplementary Figures 1-7, Supplementary Tables 1-2 and Supplementary References [file ncomms12200-s1.pdf]

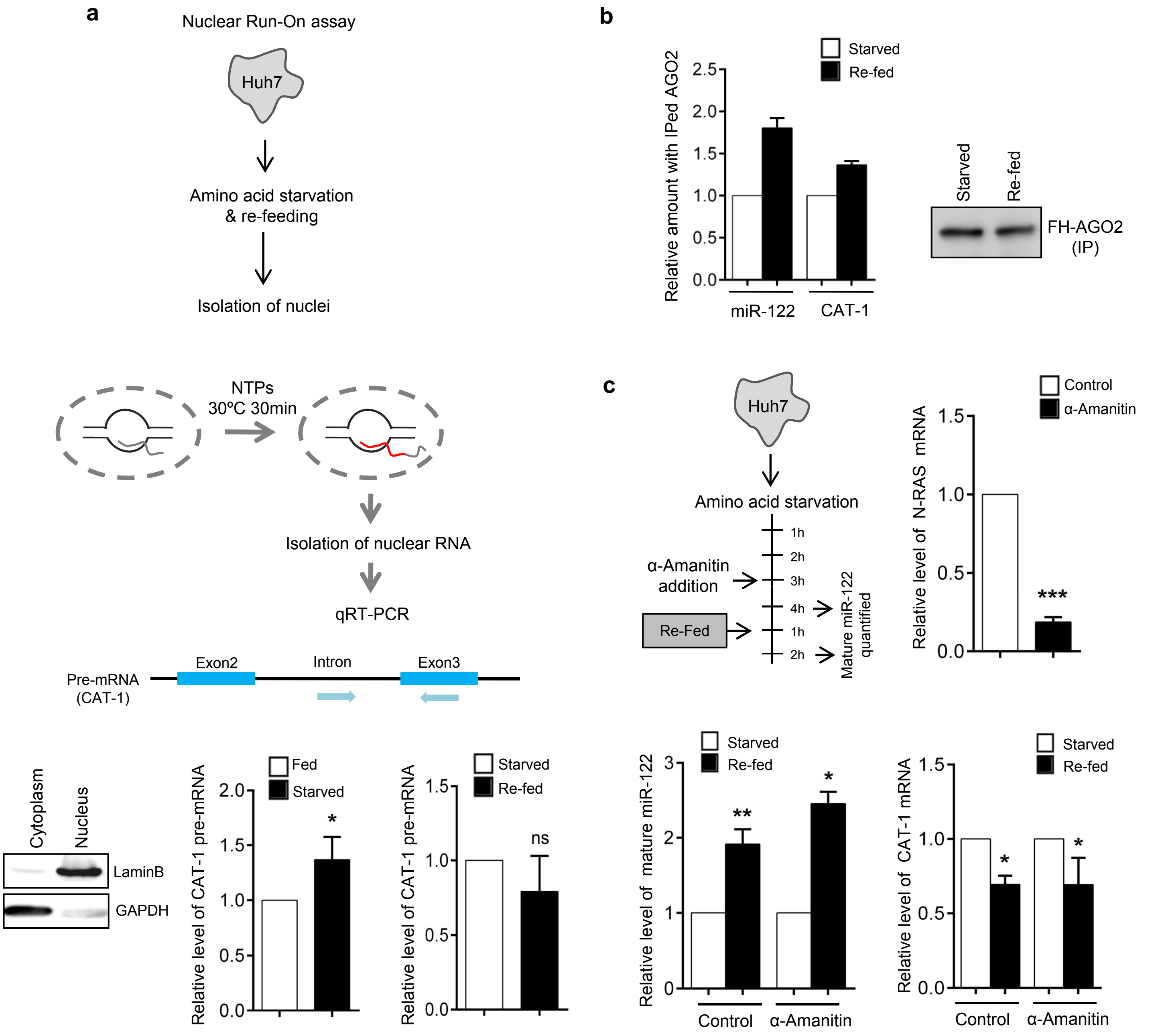

Supplementary Figure 1 | Increase in miR-122 upon re-feeding is post-transcriptional

(**a**) Transcription of CAT-1 mRNA is not downregulated significantly upon re-feeding of starved Huh7 cells. Nuclear Run-On assay was performed with nuclei isolated from Fed, Starved and Re-fed cells. Nascent CAT-1 mRNA was detected using primers spanning intron 2 and exon 3 and normalized to nuclear β-Actin pre-mRNA levels. Western blot using equal amount of protein shows efficient isolation of nucleus from Huh7 cells as indicated by the distribution of nuclear marker protein LaminB and cytoplasmic marker GAPDH.

(**b**) Increased miR-122 is AGO2 associated in Re-fed Huh7 cells. Huh7 cells transiently transfected with FH-AGO2 were starved and subsequently fed as depicted earlier and AGO2 associated miR-122 and CAT-1 mRNA levels was measured and normalized values obtained from qPCR against immunoprecipitated AGO2 were plotted. One representative blot of immunoprcipitated AGO2 has been shown.

(**c**) α-Amanitin mediated transcriptional blockage does not affect the increase in mature miR-122 levels in Re-fed cells. Scheme of the experiment has been shown. Transcriptional blockage by α-Amanitin (10 µg/ml) was confirmed by measuring N-Ras mRNA levels. CAT-1 mRNA also did not show any change upon transcriptional blockage reconfirming that CAT-1 decrease upon re-feeding was not transcriptional. Quantification of mature miR-122 and CAT-1 mRNA levels in control and α-Amanitin treated cells was done by real time PCR.

Paired two-tailed Student's t tests were used for all comparisons. $p < 0.05$ (*); $p < 0.01$ (**); $p < 0.001$(***). For (a) and (c) *n*=3. For (b) *n*=2. Error bars represent S.D.

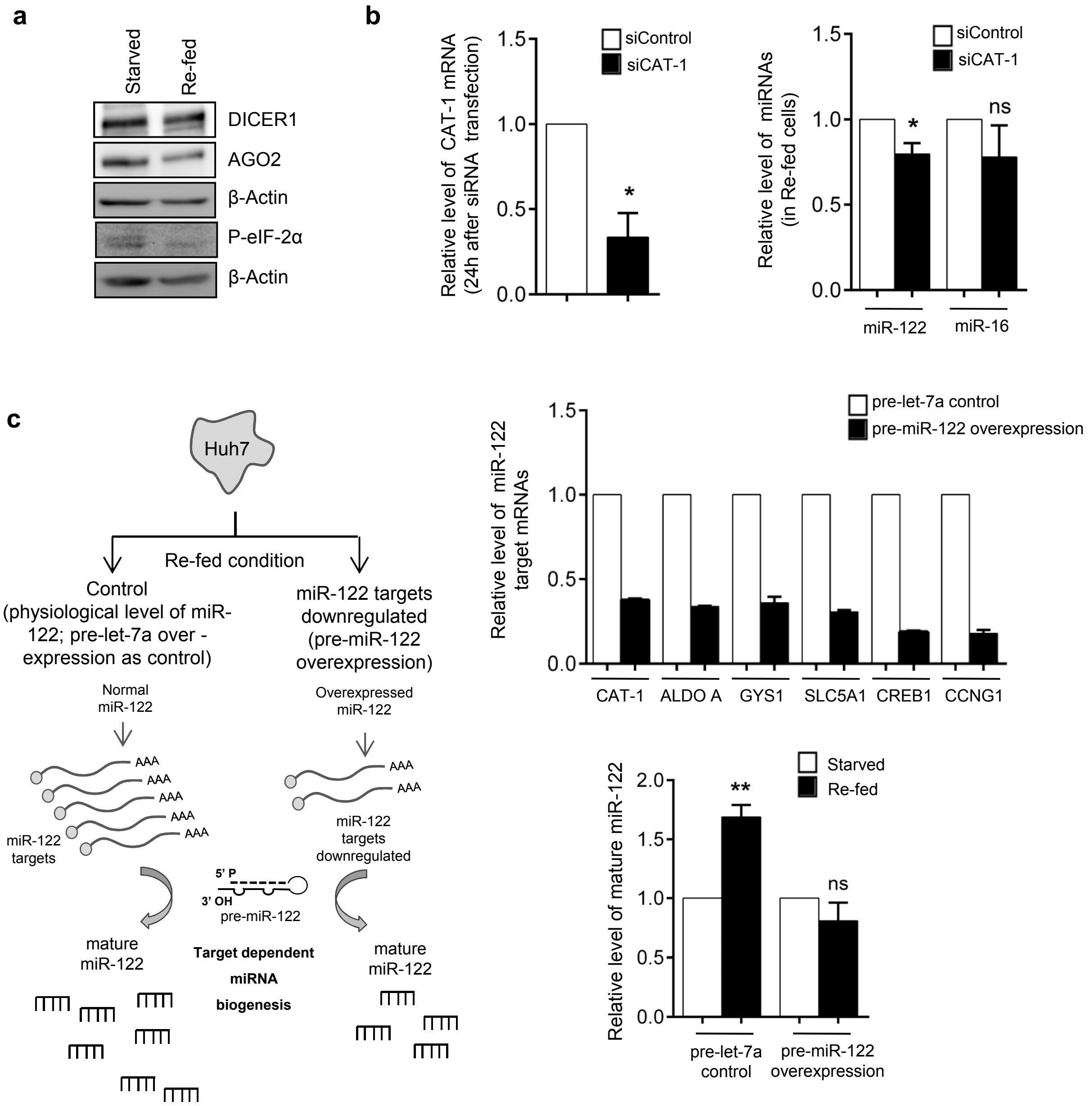

Supplementary Figure 2 | miR-122 biogenesis in re-fed cell depends on DICER1 and miR-122 target availability

(**a**) Feeding Huh7 cells after 4h starvation does not affect DICER1 or AGO2 protein levels significantly. Western blotting for endogenous DICER1 and AGO2 levels are shown. Phospho-eIF-2α is a marker of cellular stress.

(**b**) Knockdown of CAT-1 mRNA partially prevents target driven biogenesis of mature miR-122 upon re-feeding. Left panel shows siRNA mediated knockdown of CAT-1 mRNA 24h after transfection. 24h after transfection cells were starved and subsequently re-fed and mature miR-122 and miR-16 levels quantified.

(**c**) Downregulation of miR-122 target mRNAs prevents target-driven miR-122 biogenesis upon relief of amino acid starvation induced stress. Scheme of the experiment is outlined in left panel. Huh7 cells overexpressing pre-miR-122 or pre-let-7a (control) were subjected to amino acid starvation for 4h followed by re-feeding of cells for 2h. Synthetic pre-miRNA from Life Technologies were used for overexpression at a concentration of 25nM. miR-122 overexpression was confirmed by measuring levels of six different miR-122 target mRNAs including CAT-1. Mature miR-122 levels were quantified by real-time PCR.

For (b) *n*=3 and *n*=2 for (c). Paired two-tailed Student's t tests were used for all comparisons. $p < 0.05$ (*); $p < 0.01$ (**); $p < 0.001$(***). Error bars represent S.D.

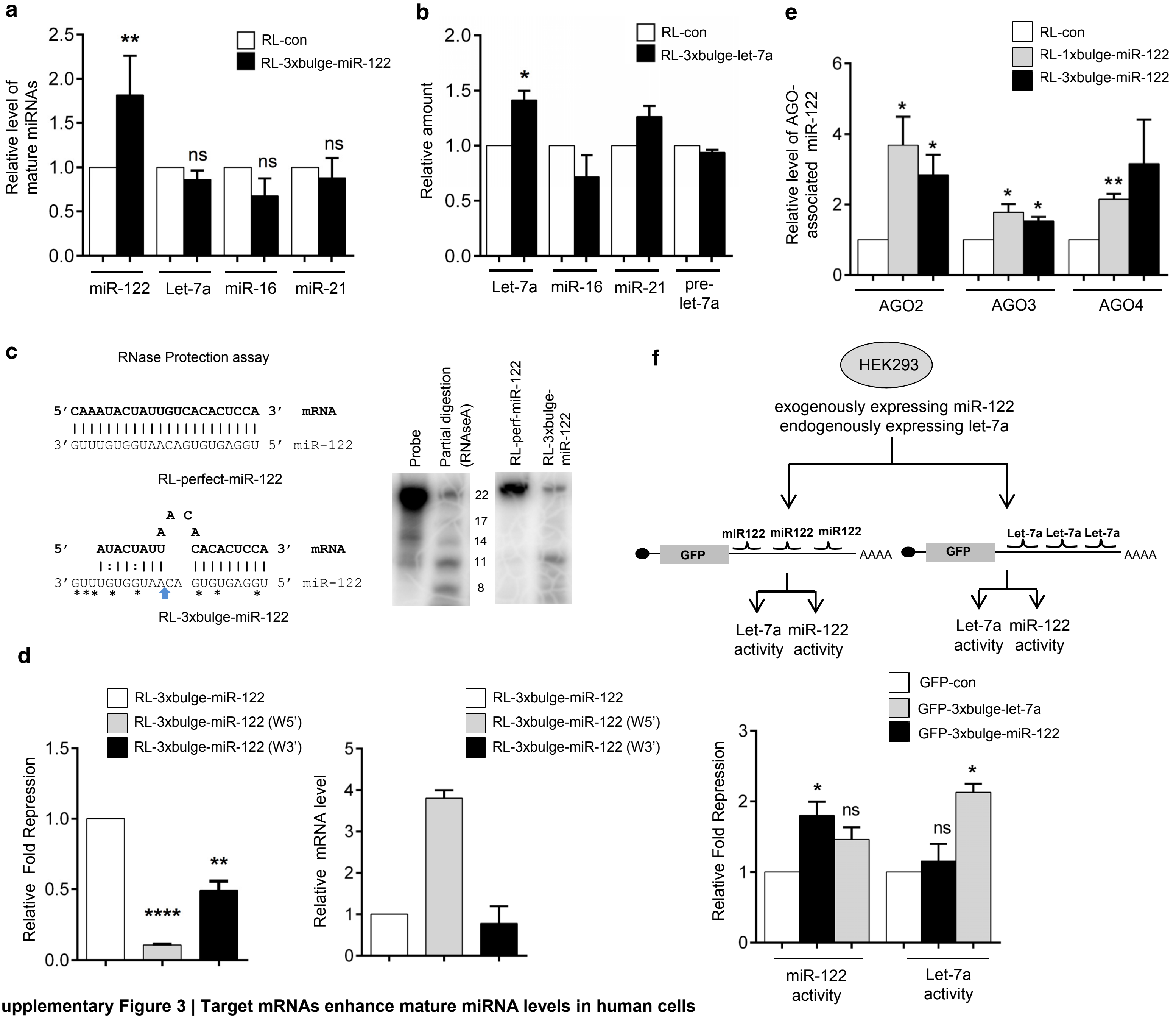

Supplementary Figure 3 | Target mRNAs enhance mature miRNA levels in human cells

(**a**) Effect of expression of miR-122 substrate on target and non-target miRNA levels. Let-7a, miR-16 and miR-21 levels were quantified from HEK293 cells expressing miR-122 target and pre-miR-122.

(**b**) Target dependent miRNA increase of let-7a in HEK293 cells. Mature miRNAs and pre-let-7a miRNA levels measured by qPCR in cells expressing RL-con or RL-3xbulge-let-7a target mRNAs. Non-specific miRNAs, miR-16 and miR-21 were unchanged.

(**c**) The 3'half of miRNA binds with target mRNA. RNase protection assay was done with radiolabelled synthetic miR-122 in presence of target mRNAs. The blue arrow indicates the Cytosine at position 11 that was cleaved by RNAse A to generate the 11nt band and other potential cleavage sites of RNaseA has been marked with *. Original intact blot in Supplementary fig. 7.

(**d**) Effect of modification of 5' or 3' sequence on RL3xbulge-miR-122 on repression and mRNA levels.

(**e**) miR-122 association with Argonautes in presence of target mRNA. FH-AGO2 and HA-tagged AGO3 and 4 were expressed in HEK293 cells along with target mRNAs and pre-miR-122 and immunoprecipitated AGOs were used for miR-122 quantification from the respective AGO expressing cells. Real-time PCR based quantification of miR-122 association with AGO2, AGO3 and AGO4 in presence of target mRNAs.

(**f**) Repressive activity of miRNP is enhanced in presence of cognate mRNA. miR-122 and let-7a activity were measured in HEK293 cells expressing pre-miR-122 and GFP-3xbulge-miR-122 (miR-122 target) or GFP-con, by luciferase activity assay. Similar assay was carried out with cells expressing pre-miR-122 with GFP-3xbulge-let-7a (let-7a target) or GFP-con. In both cases, GFP-encoding target mRNAs were transfected along with pre-miR-122, Firefly Luciferase (FF)-encoding plasmid and RL-con or RL-3xbulge-miR-122 reporters were used for measuring repressive activity of the enhanced miRNPs formed. Scheme of the experiment is shown in the flowchart.

Paired two-tailed Student's t tests were used for all comparisons. $p < 0.05$ (*); $p < 0.01$ (**); $p < 0.001$(***). In (a-f) values are means from at least three independent transfections. Error bars represent S.D.

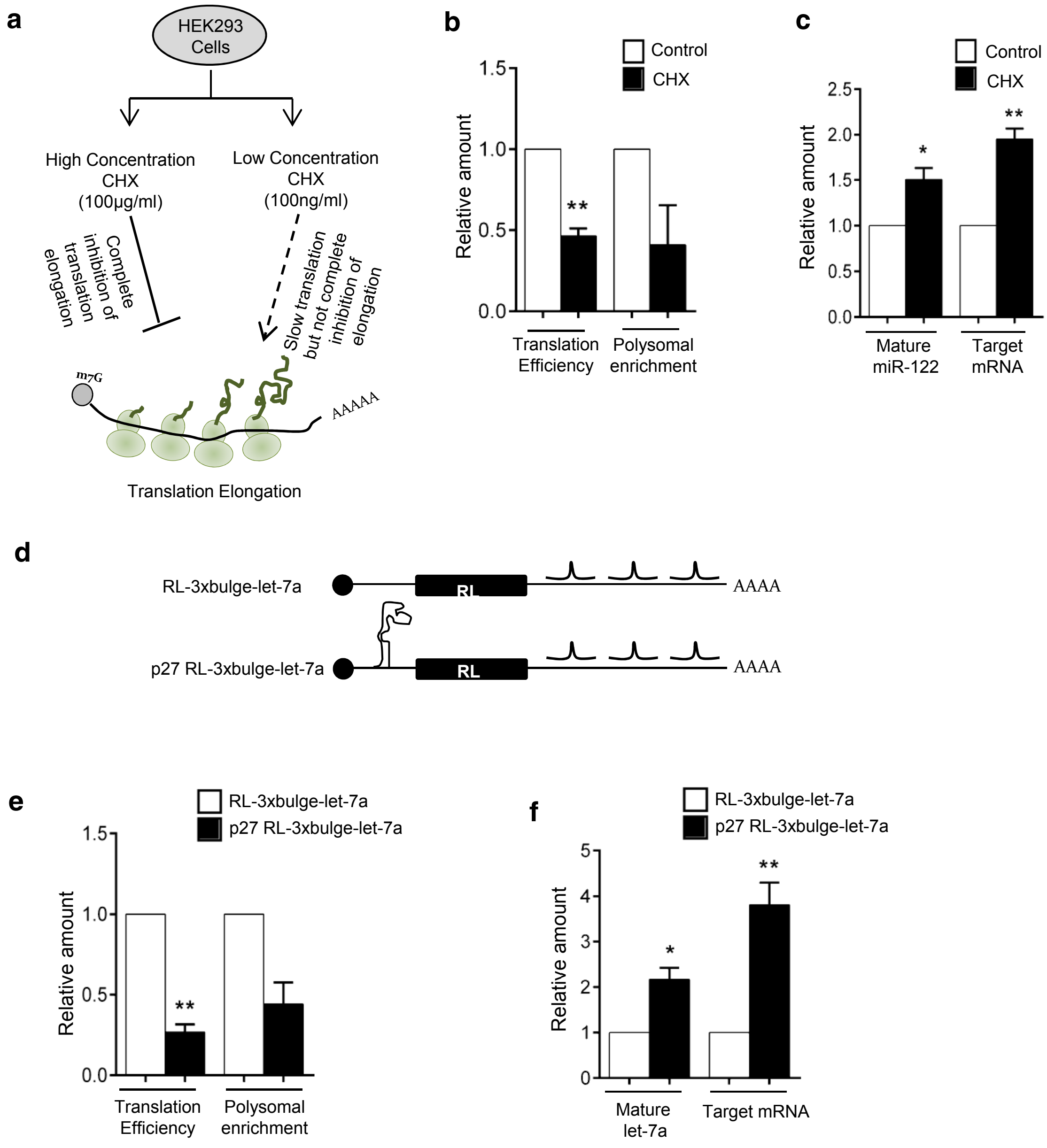

Supplementary Figure 4 | Effect of translation of target mRNA on target driven miRNA increase

(**a**) Schematic outline of experiment has been shown.

(**b-c**) Effect of low concentration of cycloheximide on target driven miRNA production. HEK293 cells expressing RL-con were subjected to 100ng/ml CHX overnight and luciferase activity measured. The luciferase activity has then been normalized to the mRNA levels quantified by qPCR. The luciferase activity of RL-con per unit mRNA has been plotted as relative translation efficiency. Relative polysomal enrichment of RL-3xbulge-miR-122 mRNA has also been plotted (**b**). Effect of CHX treatment on mature miR-122 and target mRNA level in HEK293 cells (**c**).

(**d**) Diagrammatic representation of the p27 RL-3xbulge-let-7a mRNA with p27 secondary structure element in its 5’UTR.

(**e-f**) Effect of *p27* structural element on target-driven miRNA production. Firefly luciferase (FL) normalized Renilla luciferase (RL) activity obtained with RL-con mRNA (with or without p27 element) was measured and normalized to the corresponding mRNA level. The luciferase activity per unit mRNA have been represented as the translation efficiency. Relative polysomal enrichment of the mRNAs was calculated by normalizing polysomal mRNA level with total cellular level (**e**). Mature let-7a and target mRNA levels have been quantified by qPCR from HEK293 cells expressing either RL-3xbulge-let-7a or p27-RL-3xbulge-let-7a (**f**).

Paired two-tailed Student’s t tests were used for all comparisons. $p < 0.05$ (*); $p < 0.01$ (**); $p < 0.001$(***). In (b-c) and (e-f) *n*=3. Error bars represent S.D.

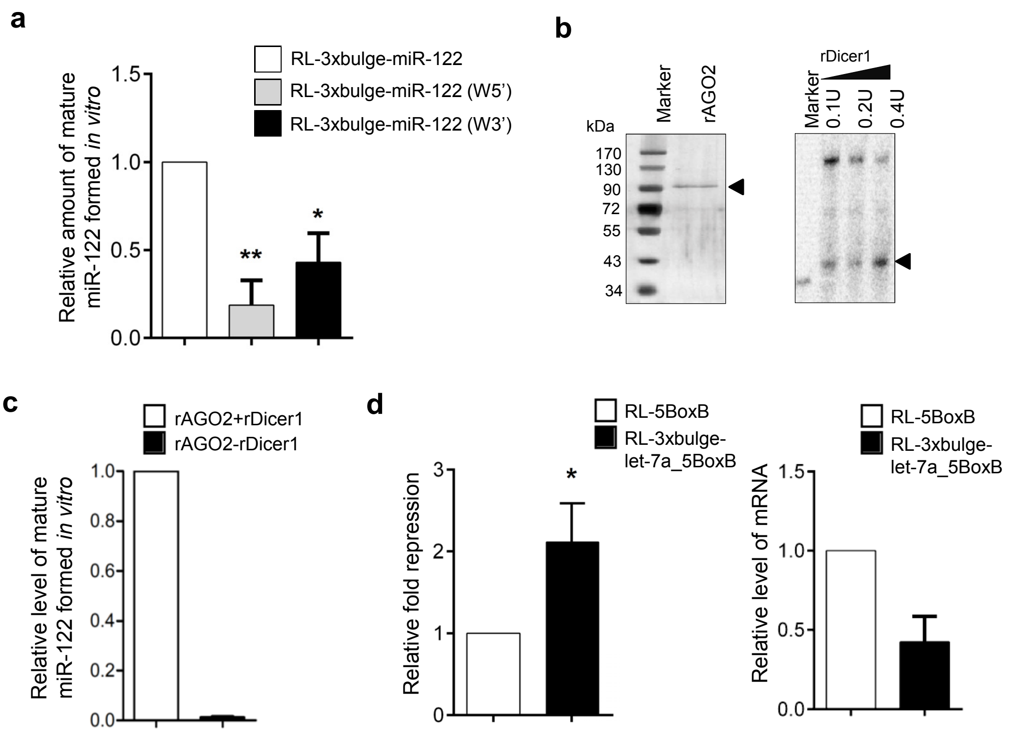

Supplementary Figure 5 | Increased processivity of AGO2-associated DICER1 enhances miRNA production in presence of target mRNA

(**a**) *in vitro* pre-miRNA processing assay with RL-3xbulge-miR-122 and mutant target mRNAs .

(**b**) Silver staining of rAGO2 confirmed its purity and absence of DICER1 contamination (left). Pre-miRNA processing activity of rDICER1 has been verified (right). Arrowheads mark the exact rAGO2 band in the silver-stained gel (left) and the mature miR-122 formed (right).

(**c**) *In vitro* pre-miR-122 processing assay in presence and absence of rDICER1 further confirmed the purity of rAGO2.

(**d**) Presence of BoxB secondary structures does not affect the efficiency of let-7a sites on RL-3xbulge-let7a_5BoxB. Fold repression and mRNA level of RL-3xbulge-let7a_5BoxB have been plotted taking RL-5BoxB as control.

Paired two-tailed Student's t tests were used for all comparisons. $p < 0.05$ (*); $p < 0.01$ (**); $p < 0.001$(***). In (a) *n*=3. For (c-d) *n*>=2. Error bars represent S.D.

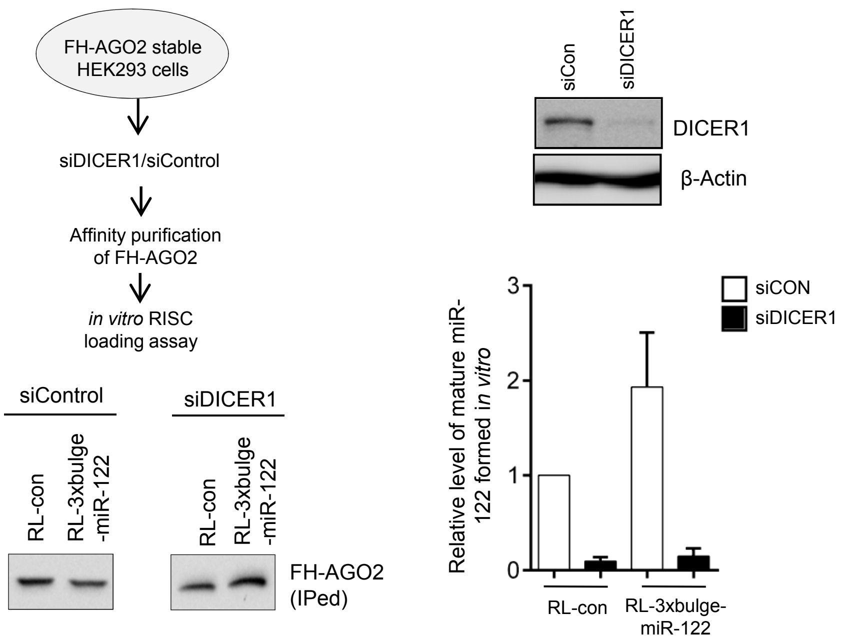

Supplementary Figure 6 | Role of DICER1 in target driven miRNA biogenesis

Effect of DICER1 knockdown on miRNA biogenesis. AGO2 associated mature miR-122 levels have been calculated by normalizing miR-122 formed with immunopurified AGO2. Western blot data for immunoprecipitated FH-AGO2 from one set of experiment has been shown. Western blot for DICER1 shows efficient knockdown in siDICER1 transfected HEK293 cells. Relative effect of target mRNA on miRNA-association of FH-AGO2 isolated either from siControl of siDICER1 treated cells were measured and plotted.

Values are means from at least two biological replicates. Error bars represent S.D.

Figure 1g

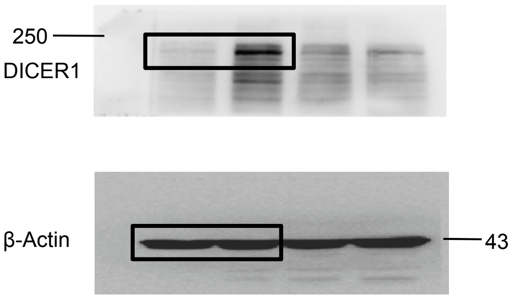

Figure 5a

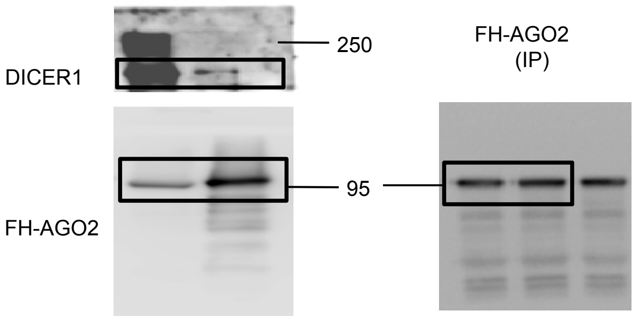

Figure 5c

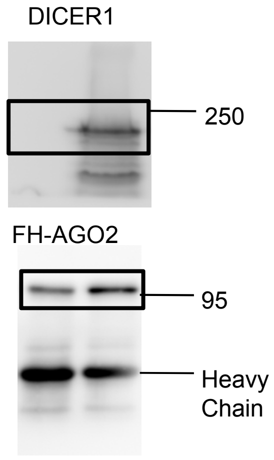

Supplementary Figure 1a

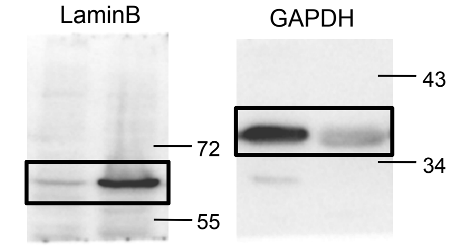

Supplementary Figure 1b

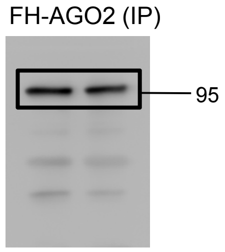

Supplementary Figure 2a

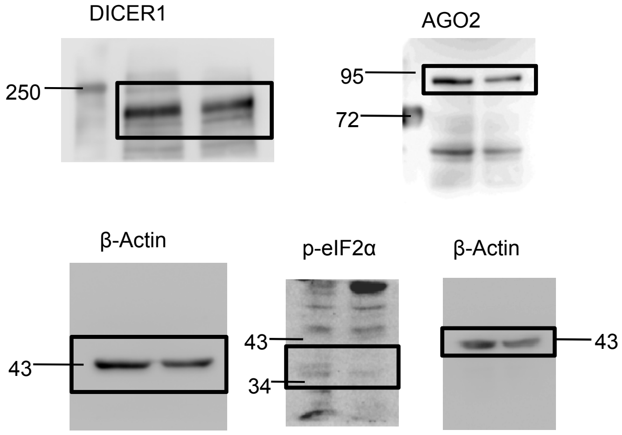

Supplementary Figure 5

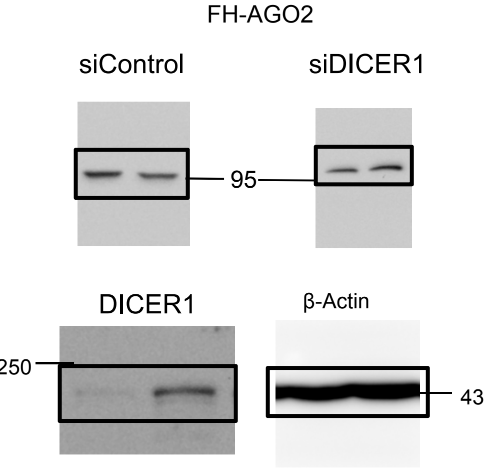

Supplementary Figure 6b

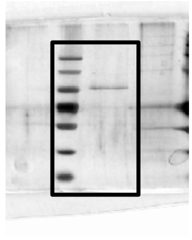

Supplementary Figure 7 | Original images of blots and autoradiographs (Western blots)

Figure 1c

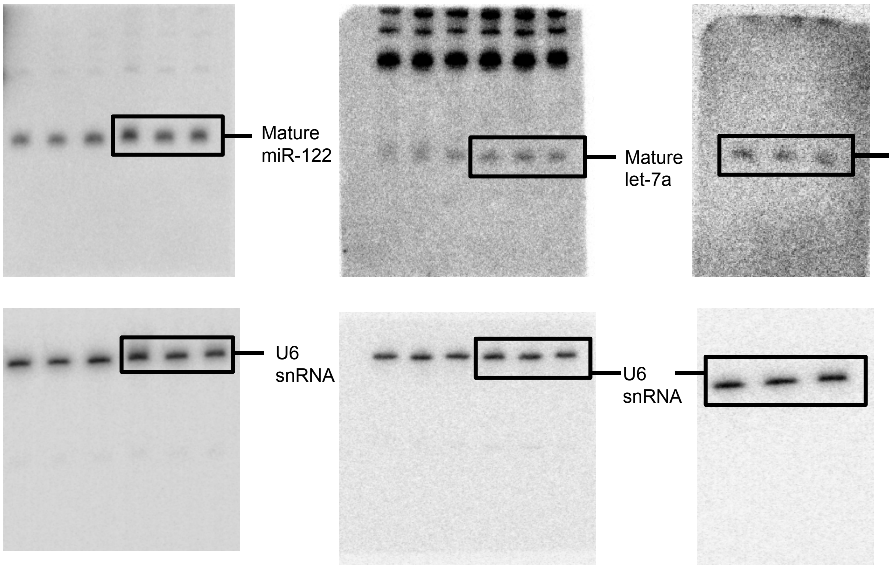

Figure 1f

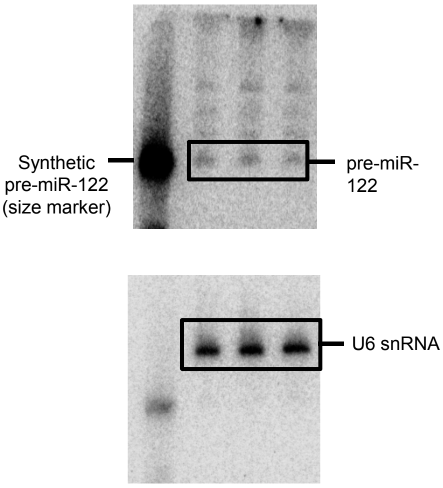

Figure 2b

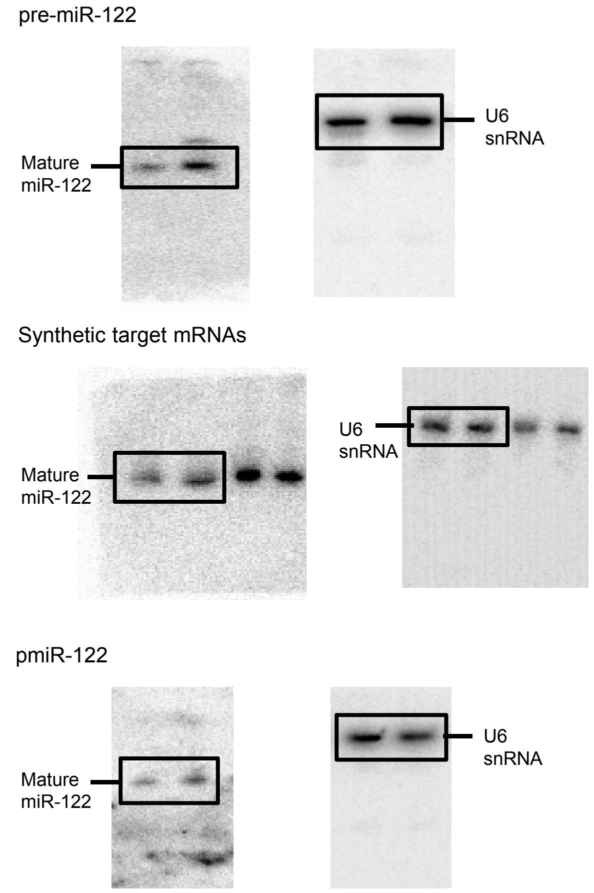

Figure 2e

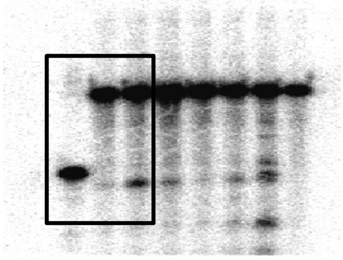

Figure 5a

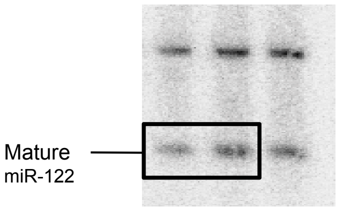

Supplementary Figure 3c

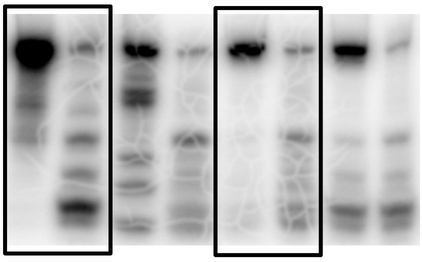

Supplementary Figure 6b

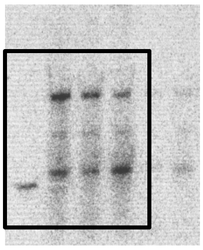

Supplementary Figure 7 | Original images of blots (Northern) and autoradiographs

Supplementary Table 1: List of Plasmids

Name of plasmids	Reference/ Source	Plasmid description
pRL-con	From Witold Fillipowicz [1]	Humanized Renilla Luciferase coding region.
pRL-3xbulge-miR-122	From Witold Fillipowicz [2]	Three miR-122 binding sites downstream of Renilla Luciferase (RL) coding region.
pRL-3xbulge-let-7a	From Witold Fillipowicz [1]	Three let-7a binding sites downstream of Renilla Luciferase (RL) coding region
pRL-3xbulge-mut	From Witold Fillipowicz [1]	Three mutated let-7a binding sites downstream of Renilla Luciferase (RL) coding region
pRL-1xbulge-miR-122		One miR-122 binding sites downstream of Renilla Luciferase (RL) coding region within XbaI and NotI sites
pGL3FF	From Promega	Firefly Luciferase (FL) under SV40 promoter
pmiR-122	As described by Chang J. [3]	Plasmid encoding pre-miR-122 under a constitutive U6 promoter
p27- RL-3xbulge-let-7a		5'UTR of human p27^{Kip1} cloned upstream of RL3xbulge-let-7a in NheI site
pIRE-RL	From Witold Fillipowicz	Ferritin IRE sequence upstream of RL coding region
IRE-RL-3xbulge-miR-122		3xbulge-miR-122 sequence from RL-3xbulge-miR-122 cloned downstream of RL coding region of pIRE-RL in NheI and NotI sites.
GFP-con		Plasmid expressing GFP protein, amplified and cloned in pCI neo vector
GFP-3xbulge-miR-122		GFP replacing RL in pRL-3xbulge-miR-122
GFP-3xbulge-let-7a		GFP replacing RL in pRL-3xbulge-let-7a
pRL-3xbulge-miR-122 Weak 5'(W5')		Three miR-122 binding sites (with weak base pairing in 5' seed region) downstream of Renilla Luciferase (RL) coding region.
pRL-3xbulge-miR-122 Weak 3'(W3')		Three miR-122 binding sites (with weak base pairing in the 3' half) downstream of Renilla Luciferase (RL) coding region.
FH-AGO2	From Tom Tuschl [4]	FLAG and HA tagged human AGO2 expression plasmid
NHA-AGO3, NHA-AGO4	From Witold Fillipowicz	N-HA tagged human AGO3 or AGO4 expression plasmid
pTet-On® Advanced Vector	From Clontech	Expressing Tet-responsive reverse transactivator
pTRE-Tight-BI Vector	From Clontech	Expression plasmid containing a promoter with modified Tet-responsive element
imiR-122		Pre-miR-122 sequence cloned within pTRE-Tight-BI Vector in NheI and NotI sites
pre-miR-122 mimic	From Life technologies (PM11012)	
pre-let-7a mimic	From Life technologies (PM 10050)	

Supplementary Table 2: List of Primers

Name of Gene	Forward Primer	Reverse Primer
RL	5’ CCAAGCAAGATCATGC 3’	5’ GCTCTTGATGTACTTACCC 3’
18s rRNA	5’ TGACTCTAGATAACCTCGGG 3’	5’ GACTCATTCCAATTACAGGG 3’
Pre-miR-122	5’ AGCTGTGGAGTGTGACAATG 3’	5’ GCTATTTAGTGTGATAATGGC 3’
Pre-let-7a	5’ TGGGATGAGGTAGTAGGTTGT3’	5’ ATAGTTATCTCCCAGTGGTGGGT 3’
U6 snRNA	5’ CTCGCTTCGGCAGCACATATACT 3’	5’ ACGCTTCACGAATTTGCGTGTC 3’
CAT-1	5’ GCCGCCGGCTTGGATTCTGA 3’	5’ CCCCGAGGGCCAGATCA 3’
β-Actin	5’ CAGATCATGTTTGAGAGACCTTC 3’	5’ CCCAGGAAGGAAGGCTGGAAG 3’
GYS-1	5’ GGTGGCTAACAAGGTGGGTGGC 3’	5’ CGATCAGCCAGCGCCCGAAA 3’
ALDO A	5’ TGGACCTAGCTTGGCGCGGA 3’	5’ CCTGGGCCAGCAGGCAGTTC 3’
SLC5A1	5’ TCCTTCAATGAGGCCACC 3’	5’ TGCGGGTGAAGAGGAAGTAG 3’
CCNG1	5’ATGACTGCAAGACTAAGGGAC 3’	5’AAAGCAGCTCAGTCCAACAC 3’
CREB1	5’GAGAACCAGCAGAGTGGAG 3’	5’TTGAACTGTCTGCCCATTGG 3’
N-RAS	5’ TCATGGCGGTTCCGGGGTCT 3’	5’ TCAACACCCTGTCTGGTCTTGGC 3’
CAT-1 3’UTR	5’GGGACTGTTCTGGGATTGAA 3’	5’ GCCCTAATGACCCATCTGAA 3’
CAT-1 pre-mRNA (NRO assay)	5’CTGTCTGTGTTTCCATAATGTGAT 3’	5’ AGCCAGGACGTAGACACCAG 3’
β-Actin/ActB (NRO assay)	5’ GGCATGGGTCAGAAGGATT 3’	5’ CACACGCAGCTCATTGTAGA 3’
RL-3xbulge-miR-122 3’UTR	5’ GAGCAGTAATTCTAGAGCGG 3’	Outer R.P: 5’ATTGCAGCTTATAATGGTTAC 3’<br>Inner R.P:<br>5’CTGCTCGAAGCGGCC 3’

Supplementary References

1 Pillai, R. S. *et al.* Inhibition of translational initiation by Let-7 MicroRNA in human cells. *Science* **309**, 1573-1576, doi:10.1126/science.1115079 (2005).

2 Bhattacharyya, S. N., Habermacher, R., Martine, U., Closs, E. I. & Filipowicz, W. Relief of microRNA-mediated translational repression in human cells subjected to stress. *Cell* **125**, 1111-1124, doi:10.1016/j.cell.2006.04.031 (2006).

3 Chang, J. *et al.* miR-122, a mammalian liver-specific microRNA, is processed from hcr mRNA and may downregulate the high affinity cationic amino acid transporter CAT-1. *RNA Biol* **1**, 106-113 (2004).

4 Meister, G. *et al.* Human Argonaute2 mediates RNA cleavage targeted by miRNAs and siRNAs. *Mol Cell* **15**, 185-197, doi:10.1016/j.molcel.2004.07.007 (2004).
